# Targeting platinum-resistant ovarian cancer by disrupting histone and RAD51 lactylation

**DOI:** 10.7150/thno.104858

**Published:** 2025-02-10

**Authors:** Chenggong Sun, Xiao Li, Qiuli Teng, Xihan Liu, Li Song, Helgi B. Schiöth, Huan Wu, Xinyue Ma, Zhaoyang Zhang, Changjian Qi, Haocheng Zhang, Kun Song, Qing Zhang, Beihua Kong

**Affiliations:** 1 Department of Obstetrics and Gynecology, Qilu Hospital of Shandong University, Jinan, Shandong, 250012, PR China.; 2 Gynecology Oncology Key Laboratory, Qilu Hospital of Shandong University, Jinan, Shandong, 250012, PR China.; 3 Division of Gynecology Oncology, Qilu Hospital of Shandong University, Jinan, Shandong, 250012, PR China.; 4 Department of Surgical Sciences, Functional Pharmacology and Neuroscience, Uppsala University, 751 24 Uppsala, Sweden.

**Keywords:** ovarian cancer, RAD51, lactylation, platinum resistance, HR repair

## Abstract

**Rationale:** Ovarian cancer is a highly lethal gynecological malignancy with common platinum resistance. Lactylation is involved in multiple biological processes. Thus, we explored the role of histone and non-histone lactylation in platinum resistance, providing a potential therapeutic target to overcome platinum resistance in ovarian cancer.

**Methods:** We utilized gene set enrichment analysis to investigate lactylation-related pathway alterations between platinum-resistant and platinum-sensitive patients from the TCGA cohort. Differential expression of H3K9la was demonstrated using Western blotting and immunohistochemistry. Progression-free and overall survival were determined using a log-rank test. Drug response to cisplatin was evaluated by CCK8, apoptosis flow cytometry, and clonogenic assays *in vitro*. ChIP-seq and ChIP-qPCR assays were performed to identify downstream targets of H3K9la, which was further confirmed by qRT-PCR. LC-MS/MS was conducted to identify specific lactylation sites for RAD51. Co-IP was used to reveal the interaction between GCN5 and H3K9la or RAD51la. Cell line-derived and patient-derived xenograft (PDX) models of ovarian cancer were constructed for the *in vivo* experiments.

**Results:** Our study showed elevated histone lactylation, especially of H3K9la, in platinum-resistant ovarian cancer. Moreover, high H3K9la indicated platinum resistance and poor prognosis of ovarian cancer. Impairing H3K9la enhanced response to cisplatin. Mechanistically, H3K9la directly activated RAD51 and BRCA2 expression to facilitate homologous recombination (HR) repair. Furthermore, RAD51K73la enhanced HR repair and subsequently conferred cisplatin resistance. H3K9la and RAD51K73la shared the same upstream regulator, GCN5. Notably, a GCN5 inhibitor remarkably improved the tumor-killing ability of cisplatin in PDX models of ovarian cancer.

**Conclusions:** Our study demonstrated the essential role of histone and RAD51 lactylation in HR repair and platinum resistance. It also identified a potential therapeutic strategy to overcome platinum resistance and improve prognosis in ovarian cancer.

## Introduction

Ovarian cancer is the third most prevalent yet the most lethal gynecologic malignancy in women 1. In 2020, 313,959 new cases and 207,252 deaths were reported worldwide due to this disease 2. Among its various subtypes, high-grade serous ovarian cancer (HGSOC) is the most common subtype with a notable impact, accounting for 70 - 80% of ovarian cancer-related deaths 3. Over the past decades, the incidence and mortality of ovarian cancer have been consistently declining, accompanied by an increased number of patients living with this highly lethal disease 4. Despite these positive trends, the foundational approach to first-line treatment has seen minimal evolution over the past 30 years.

Platinum-based chemotherapy combined with paclitaxel remains the primary treatment for HGSOC, with an average 5-year survival rate of less than 50% and a median overall survival (OS) of only 40.7 months 3, 5. Up to 75% of advanced HGSOC cases tend to recur and eventually develop platinum resistance, with around 20% of patients initially showing no response to frontline platinum-based treatment, considered platinum refractory 5-7. Therefore, increased awareness, research, and intervention strategies are urgently needed to overcome platinum resistance in the long-term management of patients with HGSOC.

Cisplatin (CDDP) effectively targets various cellular components, especially the DNA 8. The irreversible DNA lesions induced by CDDP typically trigger DNA damage response (DDR) and activate DNA repair 9, 10, which are crucial for maintaining genome integrity 11, 12. Genomic instability is a hallmark of most cancers 13. The Cancer Genome Atlas (TCGA) study revealed that ovarian cancer is characterized by a defective homologous recombination (HR) pathway due to genetic and epigenetic alterations in key genes 14. This underlying defect contributes to the sensitivity of cancer to platinum agents. In turn, increased awareness, research, and intervention strategies are urgently needed to overcome platinum resistance in the long-term management of patients with HGSOC restoring HR repair functions in a deficient background might represent a potential mechanism of platinum resistance. For instance, reversion mutations or intragenic deletions in mutated BRCA1/2 can restore the reading frame, resulting in a functional protein and re-establishing HR deficiency, consequently leading to resistance to CDDP 15, 16. Similarly, secondary somatic mutations in RAD51C and RAD51D, which can restore the reading frame of proteins, were observed in post-progression tumor samples from platinum-sensitive patients in the ARIEL2 clinical trial 17. RAD51 expression assays, such as RAD51 foci detection or quantitative immunohistochemistry, could help identify patients likely to respond to platinum-based treatment in preclinical models and discovery cohorts 18, 19. Overall, targeting HR repair is an essential strategy for overcoming platinum resistance, which remains the leading cause of mortality in advanced ovarian cancer.

Post-translational modifications (PTMs), a form of epigenetic modifications, are common on histone and non-histone proteins, serving as integrators of environmental signals that subsequently influence cellular responses by regulating gene function 20. Recently, lactate-derived lysine residue lactylation (Kla) has emerged as a novel PTM paradigm, playing an essential role in several physiological and pathological processes, such as immune response, embryogenesis, neural development, and tumorigenesis 21-23. Lactate, a byproduct of glycolysis, mediates histone Kla, contributing to gene activation during M1 macrophage polarization, as initially discovered by Zhang *et al.* 24. Subsequent studies revealed a major association between histone protein Kla and conditions such as lung myofibroblasts, Alzheimer's disease, myocardial infarction, and malignancies, including colorectal cancer (CRC), hepatocellular carcinoma (HCC), and ocular melanoma 21, 25-29. Further investigations demonstrated an increasing number of lactylation sites in non-histone proteins, which play vital roles in the pathogenesis of diseases, including sepsis, retinopathy, and malignancies such as gastric cancer, HCC, and CRC 30-34.

Besides identifying substrates, research into the "writers" and "erasers" essential for the occurrence and removal of Kla has progressively expanded. Histone acetyltransferase P300 has recently been identified as a lactyltransferase for histone and non-histone proteins in various cellular contexts 24. Histone deacetylases are the "erasers" of histone lactylation, specifically at the H3K18la site 35. Sirtuin family proteins Sirt2 and Sirt3 act as histone or non-histone deacetylases in neuroblastoma cells and HCC, respectively 36, 37. Given the involvement of Kla in various tumors, targeting Kla may represent an effective strategy for cancer treatment 38. However, histone or non-histone protein lactylation and its function in the tumor progression of ovarian cancer have not yet been reported.

This study highlighted the essential role of histone and RAD51 lactylation in HR repair and platinum resistance of ovarian cancer. It also revealed that GCN5 could be a novel potential “writer” of lactylation. Furthermore, we uncovered the combined effect of CDDP and GCN5 inhibitor treatment in patient-derived xenograft (PDX) models of ovarian cancer. Our findings implied that targeting lactylation is a promising therapeutic strategy to overcome platinum resistance and improve the prognosis of patients with ovarian cancer.

## Methods

### Public dataset analysis

The gene expression profile and comprehensive clinical data of ovarian cancer were obtained from the UCSC Xena database (https://xena.ucsc.edu/). Only patients with complete survival information were considered in this study (File S1). These TCGA cases were categorized into platinum-sensitive or -resistant groups based on their platinum-free interval. Gene sets related to lactylation were acquired from the MSigDB database (www.gsea-msigdb.org; Table S1). The R package cluster Profiler (v4.6.2) was utilized to perform geneset enrichment analysis (GSEA). OS/ progression-free survival (PFS) analysis of the TCGA cohort was completed using the log-rank test. The GSE206649 dataset was retrieved from the GEO database (www.ncbi.nlm.nih.gov/geo). Online databases GEPIA2 (http://gepia.cancer-pku.cn/) and Kaplan-Meier(K-M) Plotter (http://kmplot.com/) were employed in the correlation analysis of gene expression and survival curves of GCN5, respectively.

### Tissue samples and clinical data

Ovarian cancer samples and their corresponding clinical characteristics were obtained and recorded from the Department of Pathology and the Department of Obstetrics and Gynecology at the Qilu Hospital, Shandong University, respectively. The paraffin-embedded samples were utilized to generate tissue microarrays, while the frozen samples were used for protein extraction. All patients with ovarian cancer were regularly followed up for at least 5 years from the diagnosis date and categorized into either the platinum-sensitive or platinum-resistant group. All study participants provided written informed consent. The Ethics Committee of Qilu Hospital, Shandong University, approved this human research protocol (NO. KYLL-202210-052-1).

### Immunohistochemistry staining

The 4 μm paraffin sections were deparaffinized and dehydrated, followed by antigen exposure using 98 °C citrate buffer (pH 6.0) or Tris-EDTA buffer (pH 9.0). Subsequently, endogenous peroxidases were inactivated with 3% hydrogen peroxide, and nonspecific binding was blocked with 5% goat serum. Specific primary antibodies were incubated overnight at 4 °C. After adding the horseradish peroxidase-conjugated secondary antibody, the staining sections were visualized using the 3,3′-diaminobenzidine detection system (ZSGB Biotechnology, Beijing, China). The percentage and intensity of positively stained tumor cells were measured for analysis. Positively stained tumor cells were graded as: 0 (< 1% of positive cells), 1 (1% - 24% of positive cells), 2 (25% - 49% of positive cells), 3 (50% - 74% of positive cells), 4 (75% - 100% of positive cells). Staining intensities were graded into four categories: 0 for “negative”, 1 for “weak”, 2 for “moderate” and 3 for “strong”. The immunohistochemistry (IHC) score was calculated as the product of the two aforementioned scores. IHC score ≥ 7 was defined as a high expression.

### Cell lines and cell culture

A2780 and matched CDDP-resistant (A2780/DDP) cells were procured from the European Collection of Authenticated Cell Cultures. The HEK293T cells were obtained from the Chinese Academy of Sciences (Shanghai, China), whereas the OVCAR8 cell line was generously provided by Dr. Ma's laboratory at Tongji Medical College, Huazhong University of Science and Technology. SKOV3 and matched CDDP-resistant (SKOV3/DDP) cell lines were acquired from Keygen Biotech. A2780 and OVCAR8 cell lines were cultured in RPMI 1640 medium, while SKOV3 and SKOV3/DDP cell lines were cultured in McCoy's 5A medium. The HEK293T cell line was cultured in DMEM. The complete mediums were supplemented with 10% fetal bovine serum (all from Gibco) and 1% penicillin/streptomycin. SKOV3/DDP and A2780/DDP cells were additionally induced by 0.5 μg/mL CDDP (MedChemExpress, no. HY-17394). All cell lines were cultured in a humidified incubator at 37 ℃ with 5% CO2. Transwell plates with 0.4 μm pores (LABSELECT, China) were used for the co-culture system. Before the experiment, all cell lines underwent mycoplasma testing and short tandem repeat validation.

### Western blotting

Cultured cells or frozen tissues were lysed using RIPA Lysis Buffer (Beyotime, Shanghai, China) containing Protease Inhibitor Cocktail (MedChem Express, HY-K0010). Following lysis, the resulting supernatant was obtained through centrifugation, and protein concentration was determined using the BCA Assay Kit (Merck Millipore, 71285-M). Subsequently, equal amounts of protein were separated by SDS-PAGE, transferred onto PVDF membranes (Merck Millipore, ISEQ00010) using the BIO-RAD Trans-blot system, and blocked with a 5% non-fat milk solution. The membrane was incubated overnight at 4 °C in a diluted specific primary antibody buffer, followed by incubation with secondary antibodies linked to horseradish peroxidase. Band signals were visualized using an enhanced chemiluminescence detection kit (PerkinElmer, ORT2655) through Image Quant LAS 4000 (GE Healthcare Life Sciences). Tubulin and Histone3 (H3) were used as the endogenous controls for non-histone and histone proteins, respectively.

### Drug response assay

The drug response of various cell types was evaluated using the Cell Counting Kit-8 (CCK8, Beyotime, no. C0038), clonogenic formation counting, and apoptosis assays following exposure to CDDP. A total of 2000 - 3000 cells were seeded into each well of a 96-well plate and treated with CDDP or other drugs. Subsequently, 100 μL of fresh culture medium containing 10% CCK8 solution was added to each well. The absorbance at 450 nm was measured using a Varioskan Flash microplate reader (ThermoFisher Scientific). The growth curve was generated based on the relative cell viability.

In the colony formation assay, 2000 - 3000 suspending single cells were seeded in each well of 6-well plates. The pretreated cells — 2-DG (1 mM, MedChemExpress, no. HY-13966) and FX-11 (20 μM, Selleck, no. S8928) for 24 h—were subsequently exposed to CDDP for 36 h. The resulting colonies were fixed with methanol and then stained with a 0.3% crystal violet solution for counting.

Apoptotic cells were stained using the Annexin V-FITC Apoptosis Detection Kit (BD Bioscience, NJ, USA) according to the manufacturer's instructions. Subsequently, the cells were analyzed by the Beckman Cyto FLEX FCM (Beckman Coulter).

### Chromatin immunoprecipitation sequencing and analysis

DNA fragments for Chromatin immunoprecipitation sequencing (ChIP-seq) were obtained using the Magnetic ChIP Kit (Sigma-Aldrich,17-10086) according to the manufacturer's protocol. Specifically, 1 × 10^7^ of A2780/DDP cells were fixed in 1% formaldehyde to cross-link proteins with DNA. Then, the cross-linking was quenched by glycine, and the cells were subsequently lysed in a Nuclear Lysis Buffer. Afterward, the resulting DNA was sheared into 200 to 800 bp fragments through sonication (6 cycles, 18 s on and 48 s off) performed on wet ice using the Bioruptor automatic sonicator (Diagenode, Denville, NJ). DNA fragments were divided into the control or experimental (immunoprecipitation samples) groups. The immunoprecipitation sample was incubated with 8 µg H3K9la primary antibody and resuspended with Protein A/G magnetic beads overnight at 4 °C with rotation. After washing the protein A/G bead-antibody/chromatin complex, the DNA was reversely cross-linked, eluted, and purified with spin columns. Then, acquired ChIP DNA fragments were utilized for library construction and next-generation sequencing with support from Xiuyue Biol (Jinan, China). Peak calling was completed in MACS2 (v2.1.4) software, employing a default threshold of q ≤ 0.05. The bedgraph file containing the peak signals was loaded and visualized in the Integrative Genomics Viewer (IGV). The ChIP-seq peak genes underwent Gene Ontology (GO) and Kyoto Encyclopedia of Genes and Genomes (KEGG) pathway enrichment analysis, accomplished using the R packages cluster Profiler (v4.6.2). The raw ChIP-seq data were deposited to the public Genbank repository database (accession no. PRJNA1104826).

### Quantitative real-time PCR and ChIP-qPCR

The total RNA of cultured cells was extracted using Trizol (Ambion, 15596018) reagent and reverse transcribed using PrimeScript RT Reagent Kit (TaKaRa, RR037A). Complimentary DNA (cDNA) was subjected to quantitative PCR (qPCR) using TB Green Premix Ex Taq (TaKaRa, RR420A) in the 7900HT Fast Real-Time PCR System (Applied Biosystems, MA, USA). β-actin was set as the internal control, and the mRNA level was normalized against β-actin using the 2^-ΔΔCT^ method. For ChIP-qPCR, 2 μl of reverse cross-linked and purified DNA sample, as described above, was added into the qPCR reaction tube and ran in the PCR System at least 42 times. The input value (%) was calculated using the formula: % input = 2^ (CT^input^ - CT^sample^) *100%. The primers used for real-time qPCR (qRT-PCR) and ChIP-qPCR are presented in Tables S4 and S5.

### Transient transfection and lentivirus infection

Specific small interfering RNA (siRNA) and negative control siRNA (siNC) were synthesized by GenePharma (Shanghai, China) according to the base sequences listed in Table S6. Flag-RAD51 and Myc-GCN5 overexpression plasmids were provided by Boshang Biotechnology (Shandong, China). The Flag-RAD51 K40 or K73 site-specific mutant plasmid was designed and synthesized by Boshang Biotechnology (Shandong, China), in which the lysine (K) was replaced by arginine (R). Both siRNA and plasmids were transiently transfected into corresponding cells using Lipofectamine 2000 (Invitrogen, no.11668-019) according to the manufacturer's protocol.

The open reading frame of GCN5 was cloned and inserted into the pCMV overexpression vector (OriGene, USA). The short hairpin RNA oligos targeting GCN5 (shGCN5 sequence: 5'-CGTGCTGTCACCTCGAATGA-3') or RAD51 (shRAD51 sequence: 5'-GCTAAGACTAACTCAAGATAA-3') were constructed and cloned into the pLKO.1 vector (Sigma-Aldrich, USA). The lentivirus package system consisting of psPAX2 (Addgene, plasmid #12260) and pMD2.G (Addgene, plasmid #12259) was used to produce lentivirus particles with gene overexpression or knockdown in HEK293T cells. Cells were infected through lentivirus solution and selected with puromycin (Sigma-Aldrich, no. P7130) to obtain stably transfected multiple colonies.

### Co-Immunoprecipitation (co-IP)

The protein was extracted from treated cells by the immunoprecipitation lysis buffer (Beyotime, no. P0013) and precipitated using the specific primary antibodies or anti-Flag antibodies overnight at 4 °C with rotation. Protein A/G beads (Beyotime, no. P2177) were added to the lysates and the tube was incubated at 4 °C for 2 h with rotation. Immunoprecipitates were washed and denatured in boiling 1% SDS buffer. The elutes were assayed by Western blotting as described above.

### Antibodies

Table S8 lists the reagents and antibodies used in this study.

### *In vivo* experiments

In the tumor formation assay, 5 × 10^6^ shGCN5 or pLKO.1 control OVCAR8 cells suspended in 150 μL PBS buffer were injected subcutaneously into the axilla of 4-week-old female BALB/c nude mice (GemPharmatech Co., Nanjing, China). Two weeks later, the CDDP solution (5 mg/kg) or PBS buffer was intraperitoneally injected into the tumor-bearing mice every two days for 2 weeks. The tumor volume was measured and calculated as the length × width^2^ × 0.5 during treatment. After 2 weeks of treatment, the mice were sacrificed and the tumors were harvested to compare tumor burdens.

We constructed PDX models of ovarian cancer based on a previous study 39. Four-week-old female NCG (NOD/ShiLtJGpt-Prkdc^em26Cd52^Il2rg^em26Cd22^/Gpt) mice used for PDX models were purchased (J GemPharmatech Co., Nanjing, China). Fresh tumor tissues were collected from respective patients after obtaining written informed consent, mechanically cut into homogenate, and injected subcutaneously into the lower dorsal flank or axilla of the NCG mice. When the implanted tumor could be passaged stably, isometric tumor tissue homogenate was implanted in new recipient NCG mice. After two weeks, the mice were randomly assigned to three groups: PBS + DMSO, CDDP (5 mg/kg) + DMSO, and CDDP + MB-3 (MedChemExpress, no. HY-129039, 5 mg/kg) groups. The reagents were intraperitoneally administered every 2 days for 1 month. Then, tumor growth was monitored as described above. Nude and NCG mice were housed in a specific pathogen - free facility. The Ethics Committee of Qilu Hospital, Shandong University approved and monitored all animal experiments (no. DWLL-2022-100).

### Immunofluorescence (IF) staining and co-localization

The A2780 or OVCAR8 cells pre-seeded on glass coverslips were fixed with 4% paraformaldehyde and permeabilized with 0.1% TritonX-100 (Beyotime, P0096). Subsequently, 5% BSA solution was added to block non-specific binding sites. The coverslips were mixed with primary antibody overnight at 4 °C followed by multicolor immunofluorescence staining using the Treble-Fluorescence Immunohistochemistry Mouse/Rabbit Kit (Immunoway, no. RS0035) according to the manufacturer's protocol. DAPI (Abcam, no. ab104139) was used for nuclei staining for 5 min at RT. The co-localization images of the coverslips were captured using a confocal microscope SUNNY CRISM130 (Beijing, China).

### Liquid chromatography-mass spectrometry for lactylation validation

To validate the lactylation modification of the RAD51 protein, the pEnter-RAD51 plasmids were transiently transfected into HEK293T cells for 24 h. this next, the cells were incubated with L-lactate (La) (Sangon Biotech, no. A504045-0500) for an additional 24 h. The cells were then lysed using IP lysis buffer containing a protease inhibitor, and subsequently precipitated using the anti-Flag antibody. The resulting lysate was subjected to SDS-PAGE, and the gel was stained by Coomassie blue. The target gel piece (approximately 38 - 40 kDa) was excised and subjected to liquid chromatography-tandem mass spectrometry (LC-MS/MS) analysis, which was conducted using PTM Bio (Hangzhou, China).

### Molecular docking analysis

A molecular docking analysis was conducted to investigate the molecular basis underlying the interaction between GCN5 and lactyl-peptides. Briefly, the crystal structure of GCN5 was predicted by AlphaFold2 and pretreated by the Protein Preparation Wizard module in Schrödinger software. Then, the three-dimensional conformation of the lactyl-peptides was drawn using the LigPrep module. SiteMap and Receptor Grid Generation of Schrödinger were employed to identify the active pocket of GCN5. Subsequently, the lactyl-peptides were docked into the active site using the Glide extra precision (XP, Schrödinger) docking. Additionally, the XP G score and molecular mechanics-generalized Born surface area (MM/GBSA) were calculated. The binding modification was visualized by Maestro13.5.

### Direct-repeat green fluorescent protein reporter system

The pre-treated A2780 or OVCAR8 cells were co-transfected with pCBAScel (Addgene, plasmid no.#26477) and pDR-GFP (direct-repeat green fluorescent protein; Addgene, plasmid no.#26475) using Lipofectamine 2000 for 24 h. After an additional 24 h incubation, the cells were digested and suspended. The percentage of GFP^+^ cells was determined by the flow cytometry assay in Beckman CytoFLEX FCM (Beckman Coulter, USA).

### Comet assay

The comet assay was performed using the Comet Assay Kit (Beyotime, no.C2041M). Briefly, differently-treated OVCAR8 or A2780 cells were seeded into 6-well plates. After treatment with CDDP for 8 h, the cells were harvested at the indicated time and then utilized for comet assays according to the protocol. Comets were photographed using a fluorescence microscope, and tail moments were measured to quantify the degree of DNA breaks.

### Glucose uptake assay and intracellular lactate measurement

To assess glucose uptake ability, cells were exposed to 2-deoxy-2-[7-nitro-2,1,3-benzoxadiazol-4-yl] amino-D-glucose (2-NBDG) in glucose-free medium for 90 min. Then, fluorescence was measured at excitation and emission wavelengths of 485 nm and 535 nm, respectively, using the 2-NBDG Glucose Uptake Assay Kit (Abcam no. ab235976). Intracellular lactate was determined using the L-Lactate Assay Kit (Solarbio no. BC2230) following the manufacturer's protocol.

### Extracellular acidification rate (ECAR) measurement

ECAR was measured in the Seahorse XF Glycolysis Stress Test using the Seahorse XF-96 Extracellular Flux Assay Kits (Agilent Technologies no. 103020-100). Briefly, 3 × 10³ cells per well were plated overnight in a Seahorse XF-96 culture plate. Following baseline measurements, glucose, oligomycin, and 2-DG were sequentially injected through the Seahorse injection ports at designated time points. The resulting ECAR data were analyzed using the Seahorse XF-96 Wave software.

### Statistical analysis

The survival curves were generated using the log-rank test, while categorical variables were analyzed using the Chi-square test. Group data were assessed using the two-tailed student's t-test, one-way ANOVA or two-way ANOVA. The correlation analysis of gene expression was performed using Pearson r. The results of the analysis were presented as means ± standard deviation. All statistical analyses were conducted using GraphPad Prism 9 (GraphPad Software, USA). *p* < 0.05 indicated statistical significance (ns: *p* > 0.05, *: *p* ≤ 0.05, **: *p* ≤ 0.01, ***: *p* ≤ 0.001).

## Results

### Histone lactylation is associated with platinum resistance and predicts poor prognosis in ovarian cancer

**Figure 1:**
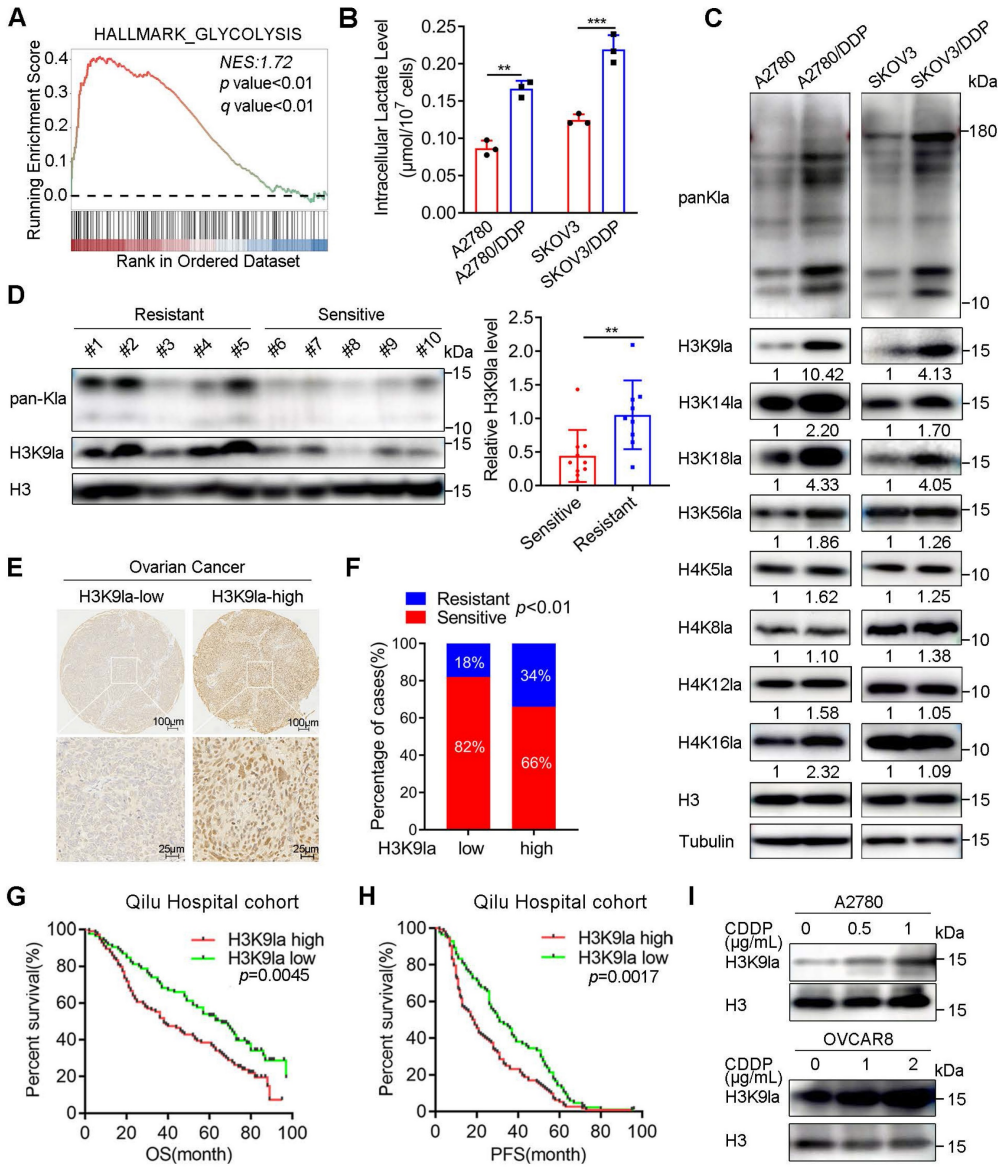
** Histone lactylation was associated with platinum resistance and predicted poor prognosis in ovarian cancer.** A. GSEA analysis of the “HALLMARK_GLYCOLYSIS” gene set between platinum-resistant and -sensitive ovarian cancer groups in the TCGA cohort. B. Intracellular lactate level in CDDP-resistant and -sensitive cells. C. Detection of pan-Kla and several site-specific histone lactylation in CDDP-resistant and control cells by Western blotting. D. Detection of pan-Kla and H3K9la levels in platinum-resistant and -sensitive samples from the Qilu Hospital cohort by Western blotting. Student's t-test was used to validate the difference in H3K9la gray values. E. Representative IHC images of H3K9la in ovarian cancer tissues. Scale bars are in the lower right corner. F. Chi-square assay to compare the platinum-resistance and -sensitive proportion in H3K9la-high and -low groups in ovarian cancer from the Qilu Hospital cohort. G and H. OS (G) and PFS (H) of H3K9la-high and H3K9la-low groups from the Qilu Hospital cohort. I. Western blotting to detect H3K9la alteration in A2780 and OVCAR8 cells pretreated with CDDP for 36 h. * p < 0.05; ** p < 0.01; *** p < 0.001; ns, no significant change.

Considering that various acetyltransferases positively regulate lactylation modifications, we defined pathways associated with acetyltransferase activity and lactate production as gene sets related to lactylation (Table S1). In the GSEA analysis of the TCGA ovarian cancer cohort, the “HALLMARK_GLYCOLYSIS” gene set was significantly elevated in the platinum-resistant group compared with the sensitive group (Figure 1A, NES = 1.72, *p* < 0.01). Meanwhile, the other four lactylation-related gene sets also exhibited remarkable increasing trends, especially “GOMF_PEPTIDE_N_ACETYLTRANSFERASE_ACTIVITY” (NES = 1.68, *p* < 0.01) and “REACTOME_GLYCOLYSIS” (NES = 1.65,* p* < 0.01), suggesting an association between lactylation and platinum resistance (Figure S1A). Therefore, we detected glycolysis in CDDP-resistant cells and control cells by Seahorse XF Glycolytic Stress Assays. The ECAR measurement revealed that CDDP-resistant cells exhibited a significantly elevated overall glycolytic flux (Figure S1B and S1C), and the relative glucose uptake of CDDP-resistant cells was increased (Figure S1D). Consistently, the intracellular lactate concentration, the chief byproduct of glycolysis, was significantly upregulated in CDDP-resistant A2780/DDP and SKOV3/DDP cells compared with the matched controls (Figure 1B).

Since lactate is the precursor of lactylation, we speculated that the lactylation level differed between platinum-resistant and platinum-sensitive patients with ovarian cancer. Thus, we examined the pan-lysine lactylation (pan-Kla) level change in CDDP-resistant and sensitive cells by Western blotting and found that the lactylation modification existed in histone and non-histone proteins (Figure 1C). CDDP-resistant cells showed a markedly higher pan-Kla level with prominent signal intensity in the histone region (10 - 15 kD molecular weight) (Figure 1C). Additionally, we detected common lactyl sites of H3 or H4 with site-specific antibodies and determined H3K9 to be the most significantly increased lactyl-histone site (Figure 1C and Figure S1E). Consistent with the results observed in cell lines, pan-Kla and H3K9la levels were also increased in platinum-resistant patients with ovarian cancer collected from the Qilu Hospital, compared with platinum-sensitive patients (Figure 1D and Figure S1F).

In the Qilu Hospital cohort, the IHC-staining assay of ovarian cancer tissue microarrays determined that the percentage of platinum-resistant cases in the H3K9la-high group was much higher than that in the H3K9la-low group (Chi-square = 6.653, *p* < 0.01; Figure 1E and 1F). The clinical characteristic analysis showed that H3K9la was also correlated with the International Federation of Gynecology and Obstetrics (FIGO) stage (Table S2). H3K9la IHC score of the platinum-resistant group was higher than the platinum-sensitive group (Figure S1G).

Additionally, we investigated the effect of H3K9la level on the ovarian cancer survival rate in the Qilu Hospital cohort. The log-rank test revealed that OS and PFS of the H3K9la-high group were significantly shorter than the H3K9la-low group (OS: hazard ratio [HR] = 1.609, 95% confidence interval [CI] = 1.159 - 2.233, *p* = 0.0045; PFS: HR = 1.548, 95% CI = 1.167 - 2.052, *p* = 0.0017; Figure 1G and 1H). The increase in H3K9la level was concentration-dependent when OVCAR8 and A2780 cells were exposed to CDDP (Figure 1I). Moreover, the CDDP-resistant cells did not exhibit an increase in the H3K9la level when treated with a higher dose of CDDP, while this trend was much more evident in sensitive cells (Figure S1H). And, the H3K9la level of CDDP-sensitive cells was elevated by co-culturing with CDDP-resistant cells (Figure S1I and S1J). Overall, these results illustrated that H3K9la played a critical role in the development of platinum resistance and predicted poor prognosis in patients with ovarian cancer.

### Inhibition of glycolysis decreased H3K9la level and increased response to CDDP in ovarian cancer

**Figure 2:**
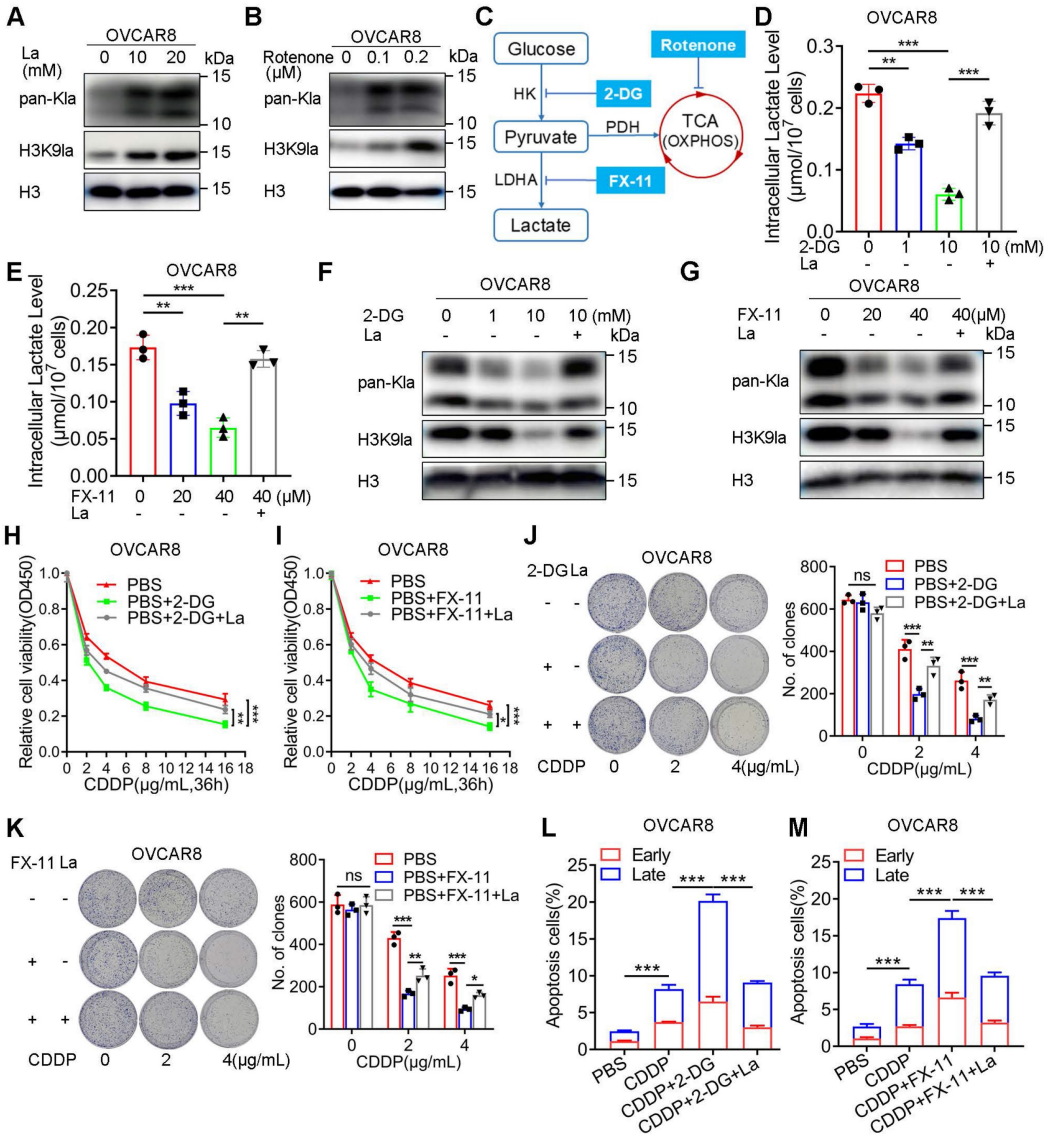
** Glycolysis inhibitors decreased the H3K9la level and increased the response to CDDP.** A and B. pan-Kla or H3K9la levels in OVCAR8 cells treated with different lactate concentrations (A) or rotenone (B) detected by Western blotting. C. Schematic diagram of glycolysis and key target inhibitors. D and E. Intracellular lactate levels in OVCAR8 cells treated with 2-DG (D), FX-11 (E), or La (10 mM). F and G. Alteration of pan-Kla or H3K9la levels regulated by 2-DG (F), FX-11 (G) and/or La (10 mM) detected by Western blotting. H and I. 2-DG (1 mM) (H) or FX-11 (20 μM) (I) impaired the relative cell viability of OVCAR8 cells exposed to CDDP, which was reversed by La (10 mM), in the CCK8 assay. J and K. Colony formation in OVCAR8 cells exposed to CDDP when cells were pretreated with 2-DG (1 mM) (J), FX-11(20 μM) (K), or La (10 mM). L and M. 2-DG (1 mM) (L) or FX-11 (20 μM) (M) increased the apoptotic cell ratio (%) induced by CDDP (2 μg/ml), which was reversed by La (10 mM) in OVCAR8 cells. * *p* < 0.05; ** *p* < 0.01; *** *p* < 0.001; ns, no significant change.

Several studies have substantiated the significant impact of glycolysis on histone lactylation due to the alteration of lactate, the substrate of lysine lactylation. To assess the effect of H3K9la on CDDP response, we demonstrated the effect of glycolysis modulation on lactylation in A2780 and OVCAR8 cells. The addition of exogenous lactate elevated the levels of pan-Kla and H3K9la in OVCAR8 or A2780 cells (Figure 2A and Figure S2A). Besides, rotenone, a mitochondrial electron transport chain complex I inhibitor, which could result in lactate accumulation in cells, could similarly elevate the pan-Kla and H3K9la levels in OVCAR8 or A2780 cells (Figure 2B and Figure S2B), indicating that both exogenous and endogenous lactate could induce lactylation in a concentration-dependent manner.

There are two key inhibitors, 2-DG and FX-11, which target hexokinase (HK) and lactate dehydrogenase (LDH), respectively, and are known to impair glycolysis (Figure 2C). The lactate assay showed that these two inhibitors could impair the intracellular lactate production in OVCAR8 and A2780 cells, which could be partially rescued by exogenous lactate (Figure 2D, 2E and Figure S2C, S2D). Consistently, Western blotting showed that both 2-DG and FX-11 could down-regulate the pan-Kla and H3K9la levels, which could be upregulated by lactate (Figure 2F, 2G and Figure S2E, S2F).

Next, we evaluated the effect of glycolysis inhibitors on the CDDP response. The relative cell viability curves illustrated that 2-DG and FX-11 reduced the relative cell viability when cells were exposed to CDDP, which could be reversed by adding lactate to the culture medium (Figure 2H, 2I, and Figure S2G, S2H). Meanwhile, the pretreatment of 2-DG or FX-11 for 24 h did not impact the cell clone formation but could significantly decrease the number of clones when cells were treated with isotonic CDDP, which could be improved by lactate (Figure 2J, 2K, Figure S2I, S2J). Furthermore, flow cytometry indicated that glycolysis inhibitors increased the percentage of CDDP-induced apoptosis in OVCAR8 or A2780 cells (early and late apoptotic cells were counted and compared), while lactate partially reversed this effect (Figure 2L, 2M, and Figure S2K, S2L, S3A, S3B). Thus, glycolysis inhibition could decrease H3K9la and improve sensitivity to CDDP.

### H3K9la enhanced HR repair by activating HR repair gene expression in ovarian cancer

**Figure 3:**
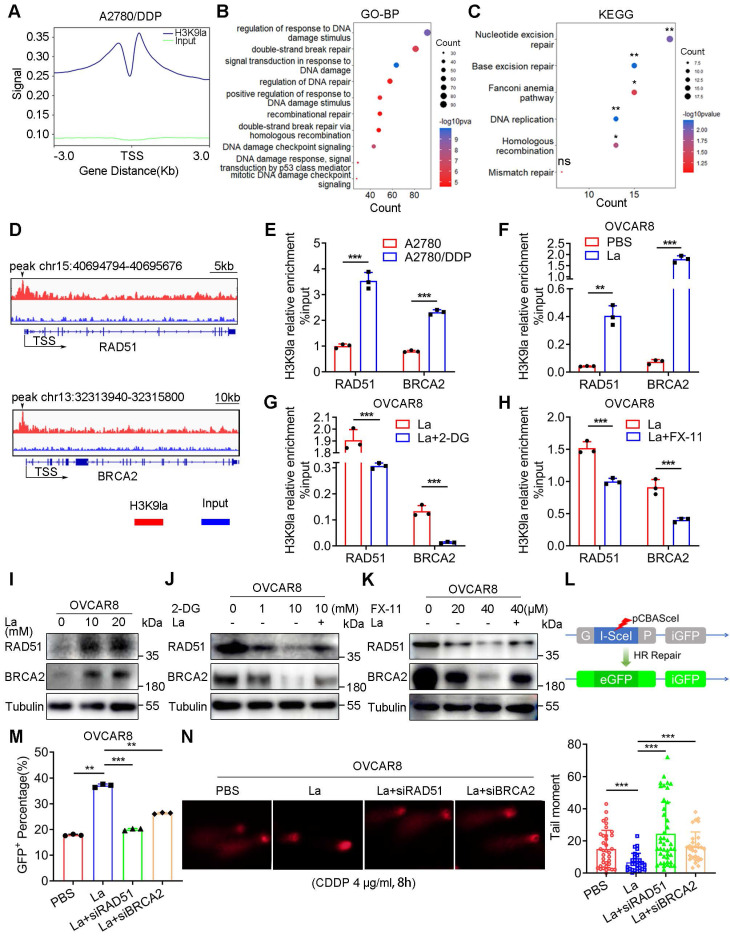
** H3K9la enhanced HR repair by activating RAD51 and BRCA2 expression.** A. ChIP intensity signal of H3K9la was distributed near the TSS. B and C. DDR-related GO (B) and KEGG (C) analysis of H3K9la-enriched downstream candidate genes. D. IGV tracks of the ChIP signal of candidate target genes. Black arrows indicated the peak regions. E. ChIP-qPCR analysis of promoters in target genes performed using anti-H3K9la in A2780 or A2780/DDP cells. F-H. ChIP-qPCR analysis of the indicated promoters in H3K9la-enriched genes in OVCAR8 cells treated with La (10 mM) (F), 2-DG (1 mM) (G), or FX-11(20 μM) (H). I. Increased RAD51 and BRCA2 protein levels by La by Western blotting. J and K. Western blotting of RAD51 and BRCA2 protein levels in OVCAR8 cells treated with 2-DG (J), FX-11 (K), or La (10 mM). L. Diagram of the DR-GFP reporter system consisting of pCBAScel and pDR-GFP plasmids. M. Fluorescence-activated cell sorting of OVCAR8 cells receiving different treatments by flow cytometry (La:10 mM). N. Comet assays (left) and quantification of tail moment (right) in OVCAR8 cells treated with10 mM La. * *p* < 0.05; **p < 0.01; ***p < 0.001; ns, no significant change.

Transcriptional activation of downstream genes is an essential molecular mechanism of histone PTMs for their specific biological functions. We performed the ChIP-seq assay using an anti-H3K9la antibody in A2780/DDP cells to identify the downstream H3K9la targets. H3K9la exhibited an enrichment signal near the transcription start site (TSS), especially the promoter regions that occupied 34.36% of the total binding peaks (Figure 3A, and Figure S4A, S4B). Additionally, we screened H3K9la-regulated downstream candidate genes more accurately with -log10 *p* > 4 and annotation with “promoter” and “protein-coding” (File S2). Restoration of the HR deficiency was reported to alleviate treatment resistance in ovarian cancer. Therefore, we created a Venn diagram between H3K9la-enriched genes and DDR genes retrieved from a previous study (File S3) 40, and observed that the H3K9la-enriched genes overlapped with 31.2% (86/276) of total DDR genes (Figure S4C) and 38.1% (8/21) of the core HR genes (Figure S4D).

Next, we retrieved all gene sets associated with DNA damage or repair pathways from the GO and KEGG databases (File S4 and Table S3). In the GO analysis, H3K9la-regulated downstream candidate genes significantly accumulated in DDR gene sets, especially HR and double-strand break repair (Figure 3B). The KEGG enrichment analysis also revealed significant accumulation in the HR pathway (Figure 3C). Among the 8 overlapped HR genes, the expression of 4 was elevated in lactate-treated OVCAR8 cells (Figure S4E). From the peak signals visualized in the IGV, we screened two H3K9la target genes, RAD51 and BRCA2, and used IGV to observe their peak signals, which were predominantly located in the TSS regions (Figure 3D). ChIP-qPCR quantitatively confirmed H3K9la enrichment in the promoter regions of the target genes in A2780/DDP cells (Figure 3E). Moreover, the ChIP-qPCR assay also confirmed that treatment with lactate or glycolysis inhibitors could enhance or impair the binding efficiency of H3K9la in promoter regions of RAD51 and BRCA2 (Figure 3F - 3H).

In the qRT-PCR assay, the RNA expression of RAD51 and BRCA2 was elevated in A2780/DDP cells (Figure S4F). Lactate effectively promoted target gene transcription by upregulating H3K9la, which could be repressed by 2-DG or FX-11 (Figure S4G - S4I). Consistently, H3K9la elevated the protein level of target genes with increasing doses of lactate or rotenone (Figure 3I and Figure S4J), while 2-DG or FX-11 suppressed H3K9la and target protein levels (Figure 3J, and Figure 3K, S4K). Besides, RAD51 and BRCA2 expression was increased in A2780 cells when co-cultured with CDDP-resistant cells (Figure S4L). The inducing effect of CDDP on BRCA2 and RAD51 in CDDP-resistant cells was not as evident as in sensitive cells (Figure S4M). Also, CDDP induced H3K9la enrichment in the promoter of RAD51 and BRCA2, thereby activating their expression, which could be partly impaired by FX-11 (Figure S4N and S4O). CDDP-induced H3K9la activated the expression of RAD51 and BRCA2, the core genes in HR repair.

Given the essential roles of RAD51 and BRCA2 in HR repair, we examined the effect of H3K9la on HR repair by a GFP-based DR-GFP reporter system (Figure 3L). Briefly, pCBAScel and pDR-GFP plasmids were co-transfected into OVCAR8 or A2780 cells to induce DNA double-strand breaks and HR repair. Flow cytometry indicated that lactate increased the percentage of HR repair-proficient (GFP^+^) cells, while the transfection with RAD51 or BRCA2 siRNA impaired the positive effect of lactylation (Figure 3M, and Figure S5A, S5B). The comet assay also showed that lactate shortened the tail moment of CDDP-treated OVCAR8 and A2780 cells, while RAD51 or BRCA2 siRNA lengthened the tail moment (Figure 3N and Figure S5C). Thus, increased H3K9la enhanced HR repair by activating the expression of H3K9la target genes in ovarian cancer.

### GCN5 positively regulated H3K9la associated with poor prognosis and platinum resistance in ovarian cancer

**Figure 4:**
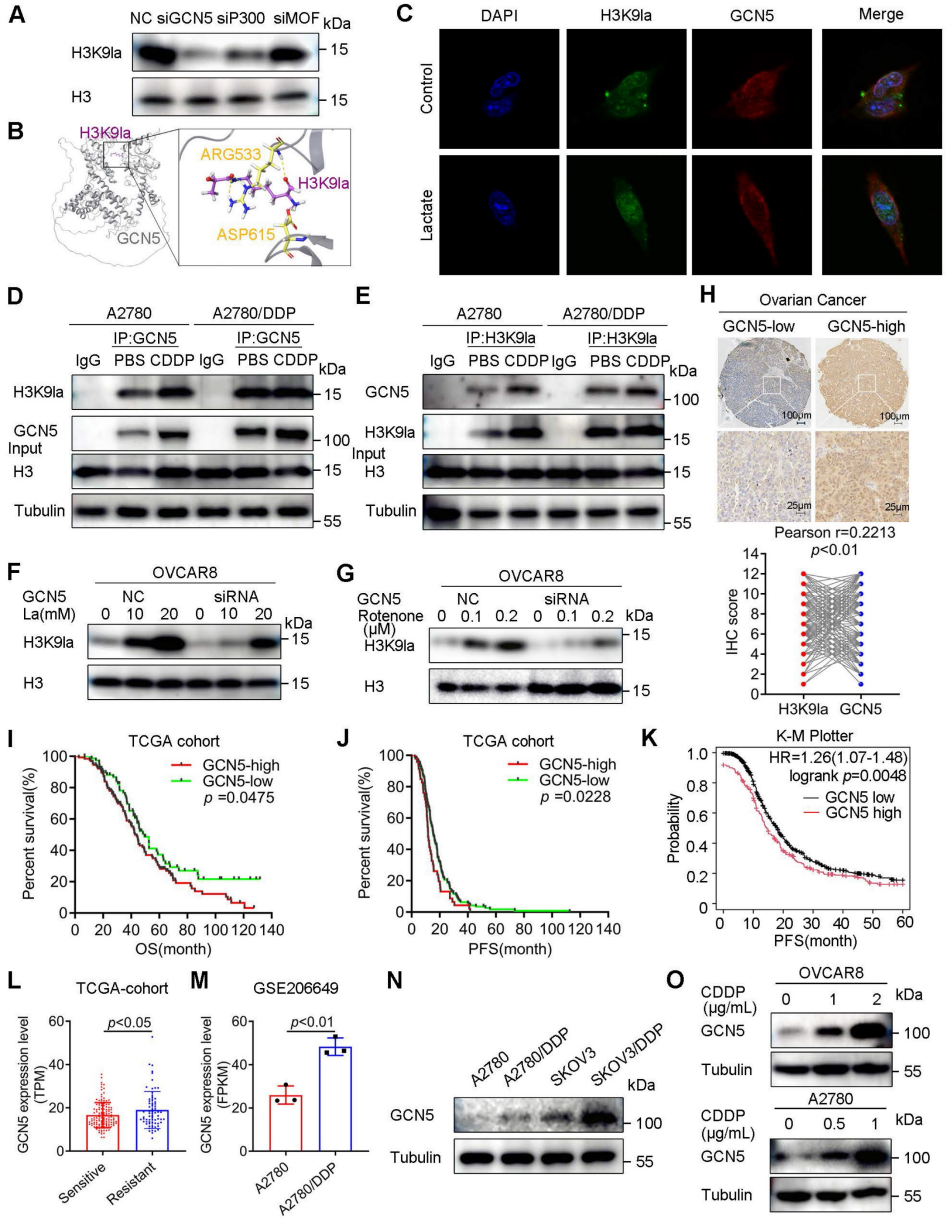
** GCN5 regulated H3K9la and was associated with poor prognosis and platinum resistance.** A. Inhibitory efficiency of siRNAs of three potential lactylation writers for H3K9la by Western blotting. B. Interaction mode between H3K9la peptide (purple) and GCN5 (gray) by the molecular docking assay. Several residues (yellow) accommodate the H3K9la group. C. Immunofluorescence co-staining for H3K9la and GCN5 in A2780 cells with/without lactate (10 mM). D and E. Western blotting of co-IP between H3K9la and GCN5 in CDDP-sensitive and -resistant cells following treatment with CDDP (1 μg/ml). F and G. Effect of GCN5 siRNA on the H3K9la level in OVCAR8 cells treated with lactate (F) or rotenone (G) by Western blotting. H. Representative IHC images of GCN5 and the Pearson correlation analysis between H3K9la and GCN5 in patients with ovarian cancer. Scale bars are in the lower right corner. I and J. Survival analysis between ovarian cancer patients with low and high GCN5 levels in the TCGA cohort, including OS (I) and PFS (J). K. K-M Plotter online analysis of PFS in ovarian cancer between low and high GCN5 groups. L. TPM level comparison of GCN5 in platinum-sensitive or -resistant patients from the TCGA cohort. M. Comparison of GCN5 expression in GSE206649 public data. N. GCN5 protein level in CDDP-resistant or control cells by Western blotting. O. GCN5 expression alteration in cells exposed to different CDDP concentrations by Western blotting. * *p* < 0.05; ** *p* < 0.01; *** *p* < 0.001; ns, no significant change.

Several histone acetyltransferases, such as P300/CBP, GCN5, and MOF, were reported as potential histone lysine lactylation writers, indicating the commonalities between acetyltransferases and lactyltransferases. Thus, the P300, MOF, and GCN5 siRNAs were transfected into ovarian cancer cells to assess the catalytic activity on H3K9la in representative HAT families. Western blots revealed that siGCN5 decreased H3K9la to the greatest extent under similar interfering efficiency (Figure S6A and Figure 4A). Then, we performed crystallographic studies by molecular docking and confirmed a stable binding model of GCN5 and H3K9la peptides (Figure 4B). IF staining demonstrated the co-localization of H3K9la and GCN5 in OVCAR8 and A2780 cells (Figure 4C and Figure S6B). Furthermore, lactate enhanced the binding of cellular GCN5 and H3K9la in the co-IP assay (Figure S6C and S6D). Besides, CDDP augmented the endogenous interaction between H3K9la and GCN5 in CDDP-sensitive cells, while this effect was impaired in CDDP-resistant cells (Figure 4D and 4E). Western blotting revealed that silencing GCN5 also attenuated H3K9la, which was induced by lactate or rotenone, in OVCAR8 or A2780 cells (Figure 4F, 4G, and Figure S6E, S6F), suggesting GCN5 as a regulator of H3K9la.

We assessed GCN5 expression in ovarian cancer tissue microarrays from the Qilu Hospital by IHC to explore its association with clinical characteristics. GCN5 had a significantly positive correlation with H3K9la (Figure 4H). Survival analysis of the TCGA cohort demonstrated that high GCN5 predicted poor prognosis of ovarian cancer, including OS and PFS (OS: HR = 1.419, 95% CI = 1.014 - 1.986, *p* = 0.0475; PFS: HR = 1.458, 95% CI = 1.008 - 2.109, *p* = 0.0228; Figure 4I and 4J). Similar results were also observed in the K-M Plotter online database (PFS: HR = 1.26, 95% CI = 1.07 - 1.48, *p* = 0.0048) and Qilu Hospital cohort (OS: HR = 1.591, 95% CI = 1.146 - 2.209, *p* = 0.006; PFS: HR = 1.334, 95% CI = 1.007 - 1.768, *p* = 0.0409; Figure 4K, and Figure S6G, S6H). Additionally, platinum-resistant ovarian cancer patients showed a higher GCN5 IHC score (Figure S6I). Consistently, GCN5 was increased in platinum-resistant patients of the TCGA cohort (Figure 4L) as well as in CDDP-resistant A2780/DDP cells (GSE206649) (Figure 4M). Western blotting also showed elevated GCN5 in A2780/DDP and SKOV3/DDP cells compared with control cells (Figure 4N). Furthermore, CDDP induced GCN5 expression dose-dependently in A2780 and OVCAR8 cells, implying the reciprocal relationship between GCN5 and CDDP resistance (Figure 4O). These results demonstrated that GCN5 was the potential “writer” of H3K9la, which predicted poor prognosis and platinum resistance of ovarian cancer.

### GCN5 conferred CDDP resistance by upregulating H3K9la and the target gene expression in ovarian cancer

**Figure 5:**
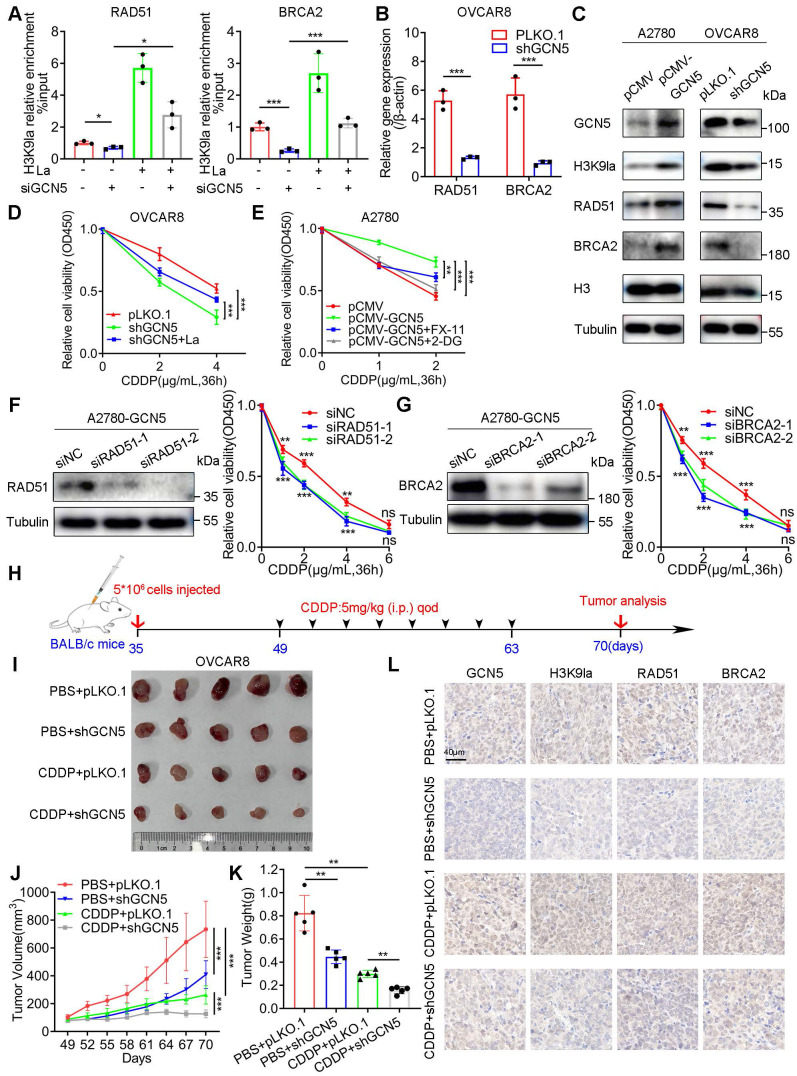
** GCN5 conferred CDDP resistance by augmenting H3K9la and its downstream target gene expression.** A. ChIP-qPCR analysis of H3K9la-targeted genes in OVCAR8 cells transfected with GCN5 siRNA and treated with lactate (10 mM) as indicated. B. qRT-PCR analysis of mRNA levels of H3K9la-targeted genes in OVCAR8 cells with shGCN5 or control pLKO.1. C. Western blotting assay for H3K9la-targeted genes in ovarian cancer cells with upregulated or downregulated GCN5. D. Effect of GCN5 on the CDDP response in OVCAR8 cells by the CCK8 assay (La: 10 mM). E. Relative cell viability in A2780 cells treated with 2-DG (1 mM) or FX-11 (20 μM) by The CCK8 assay. F and G. Interfering efficiency of siRNA for RAD51 (F) and BRCA2 (G) in A2780-pCMV-GCN5 cells by Western blotting (left). Relative cell viability by the CCK8 assay in different groups as indicated (right). H. *In vivo* tumor formation assay in BALB/c nude mice. I. Subcutaneous tumors obtained from different groups. J. Comparison of tumor volumes in different CDX groups on indicated days. K. Comparison of tumor weights of different CDX groups. L. Representative IHC-staining images of GCN5, H3K9la, RAD51, and BRCA2 from tumor tissues of Figure 5I. Scale bar: 40 μm. * p < 0.05; ** p < 0.01; *** p < 0.001; ns, no significant change.

As the potential H3K9la writer, GCN5 influenced the binding amount of H3K9la and the transcription of its downstream genes. As expected, the ChIP-qPCR assay showed that GCN5 silencing significantly reduced H3K9la binding in the promoter regions of target genes, which was then induced by lactate (Figure 5A). Western blotting revealed that GCN5 siRNA inhibited RAD51 and BRCA2 expression, which was then partly elevated by lactate-induced H3K9la (Figure S7A). Also, increased RAD51 and BRCA2 induced by Myc-GCN5 overexpression were impaired by LDH inhibitor FX-11 (Figure S7B). Moreover, the Pearson correlation test suggested that the log2 TPM (transcripts per million) value of RAD51 or BRCA2 was positively correlated with that of GCN5 as per the online GEPIA2 database (Figure S7C and S7D). Therefore, GCN5 might upregulate RAD51 and BRCA2 expression by augmenting H3K9la modification.

Next, we established stably-transfected A2780 and OVCAR8 cells to investigate whether GCN5 impacted the CDDP response by regulating H3K9la. RAD51 and BRCA2 mRNA levels were increased in GCN5-overexpressing A2780 cells but decreased in GCN5-knockdown OVCAR8 cells (Figure S7E and Figure 5B). Similar protein level changes were also observed by Western blotting (Figure 5C). In the drug response assay, GCN5 knockdown significantly impaired the relative cell viability compared with the control group *in vitro*, which was partly rescued by lactate (Figure 5D). Conversely, GCN5 overexpression decreased the CDDP response in A2780 cells, which was enhanced by glycolysis inhibitor 2-DG or FX-11 (Figure 5E).

We transiently transfected RAD51 or BRCA2 siRNAs into GCN5-overexpressing A2780 cells to illustrate the mechanism of action and determined that the CDDP-resistance conferred by GCN5 was reversed to different degrees (Figure 5F and 5G). Meanwhile, cell line-derived xenograft (CDX) models were constructed by subcutaneously injecting GCN5-knockdown OVCAR8 cells into the armpit of nude mice. Four randomized groups received CDDP or PBS intraperitoneally every other day for 2 weeks (Figure 5H) and tumor volumes and weights of the 4 treatment groups were measured and compared. Tumor analysis showed that shGCN5 or CDDP alone could inhibit tumor growth, whereas the combination of shGCN5 and CDDP exhibited a lower tumor burden and tumor volume than the single treatments (Figure 5I - 5K). Hence, GCN5 inhibition synergistically enhanced the antitumor efficacy of cisplatin *in vivo*. IHC staining of the tumor tissues revealed the regulation of downstream genes *in vivo* by GCN5. CDDP upregulated GCN5 while shGCN5 depressed H3K9la and its target gene expression, along with the enhancement of the tumor-killing effect of cisplatin (Figure 5L). Therefore, we inferred that GCN5 inhibition augmented the sensitivity to CDDP by impairing H3K9la and its target gene expression in ovarian cancer.

### RAD51 lysine lactylation enhanced HR repair and mediated CDDP resistance in ovarian cancer

**Figure 6:**
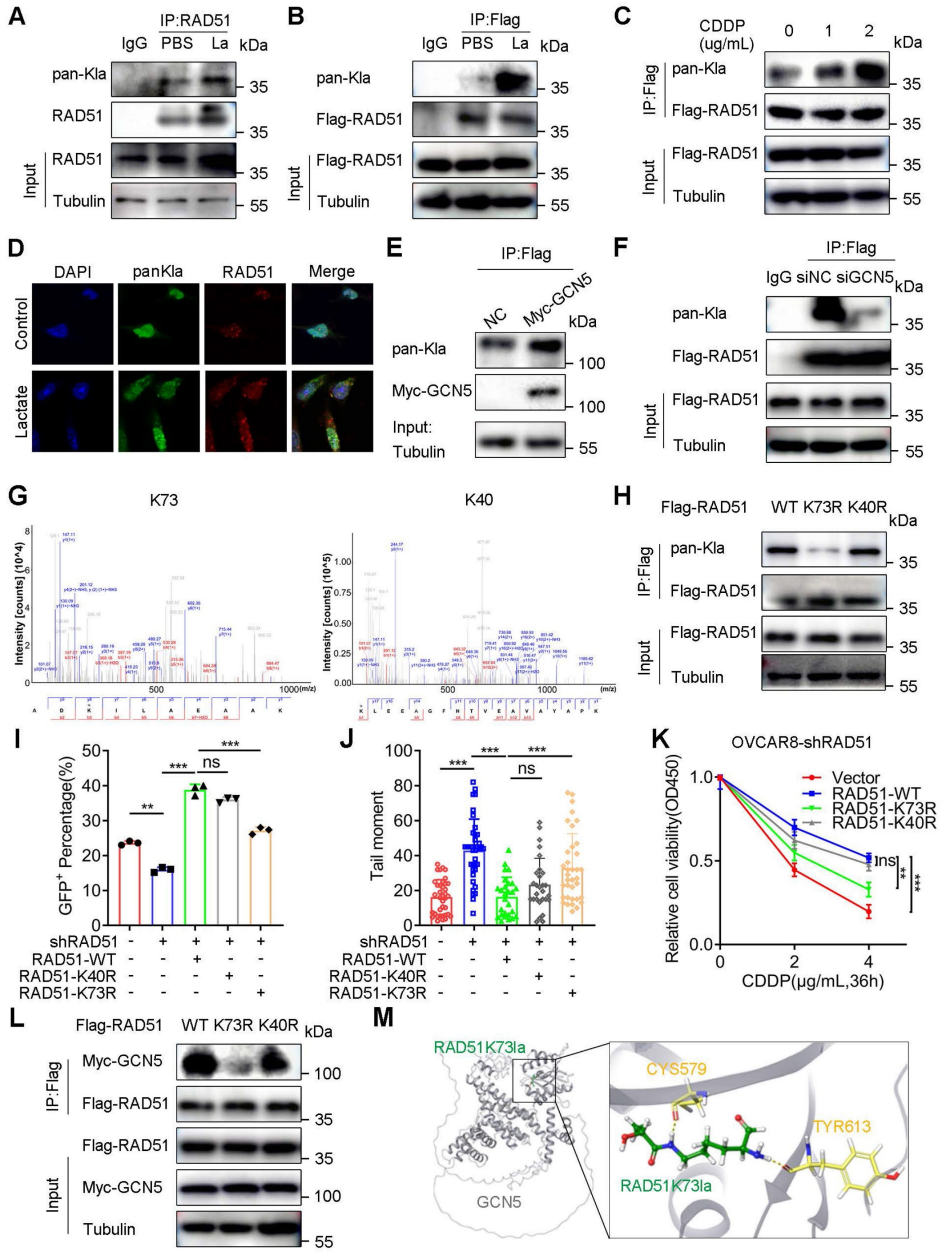
** RAD51K73la mediated CDDP resistance by enhancing HR repair, which could be regulated by GCN5.** A and B. Lactylation of endogenous (A) or exogenous (B) RAD51 protein by co-IP and Western blotting (La: 10 mM). C. Lactylation level alteration of RAD51 in cells treated with different CDDP concentrations by co-IP and Western blotting. D. Immunofluorescence co-staining for RAD51 and pan-Kla in A2780 cells with/without lactate (10 mM). E and F. Increased or decreased RAD51 lactylation with GCN5 overexpression (E) or interference (F) by co-IP and Western blotting. G. Potential RAD51 lactylation sites identified by LC-MS/MS. H. RAD51 lactylation in cells transfected with WT, K73R, or K40R vectors by co-IP and Western blotting. I. Fluorescence-activated cells were sorted by flow cytometry in OVCAR8-shRAD51 cells transfected with RAD51 WT, K73R, or K40R vectors. GFP^+^ rates were calculated and compared. J. Tail moment analysis of the treated cells from Figure 6I in comet assay. K. CCK8 assay of OVCAR8-shRAD51 cells transfected with WT, K73R, or K40R vectors in and treated with different CDDP concentrations. L. Interaction between GCN5 and RAD51 in HEK293T cells transfected with WT, K73R, or K40R RAD51 vectors by co-IP and Western blotting. M. Interaction mode between RAD51K73la peptide (green) and GCN5 (gray) in the molecular docking assay: several residues (yellow) accommodate the RAD51K73la group. * p < 0.05; ** *p* < 0.01; *** *p* < 0.001; ns, no significant change.

The lactylation of non-histone proteins and their biological function remains largely unknown. Herein, we hypothesized that H3K9la target genes might also undergo lactylation modification. To verify this hypothesis, when we applied a pan-Kla antibody for the immunoprecipitation of a potentially lactylated protein, RAD51 exhibited a more prominent Western blot band intensity than BRCA2, suggesting a relatively higher lactylation level (Figure S8A). Consistent with this observation, endogenous RAD51, which was immunoprecipitated by anti-RAD51, and exogenous RAD51, which was immunoprecipitated by anti-Flag, were both lactylated in co-IP and Western blot assays (Figure 6A and 6B), and their lactylation could be enhanced by lactate. Significantly, RAD51 lactylation was progressively elevated with the increased CDDP dose, implying a possible association with the CDDP response (Figure 6C).

Additionally, immunofluorescence co-localization of RAD51 and pan-Kla illustrated lactylation of RAD51 in A2780 or OVCAR8 cells (Figure 6D and S8B). To confirm whether GCN5 could catalyze RAD51 lactylation, we co-transfected Myc-GCN5 and Flag-RAD51 vectors into HEK293T cells and immunoprecipitated them with anti-Flag antibodies. The direct binding of exogenous RAD51 and GCN5 was evident and GCN5 overexpression significantly improved RAD51 lactylation (Figure 6E). In contrast, GCN5 siRNA sharply reduced RAD51 lactylation (Figure 6F), and the binding between endogenous RAD51 and GCN5 was also revealed (Figure S8C and S8D). These results suggested that GCN5 was the potential “writer” of RAD51 lactylation, which was similar to H3K9la.

To identify the lactylated lysine sites of RAD51, we immunoprecipitated RAD51 with the anti-Flag antibody. Subsequently, the Coomassie-stained gel section was analyzed by LC-MS/MS (Figure S8E). Two possible RAD51 lactylation sites, K73 and K40 (hereafter referred to as RAD51K73la and RAD51K40la), were identified (Figure 6G). We then constructed RAD51 K73R or K40R mutant vectors and demonstrated that RAD51 K73R mutant markedly abolished the wild-type (WT) RAD51 lactylation to a greater extent than the K40R mutant, suggesting that K73 was the most relevant site (Figure 6H). As shown in Figure S8F, RAD51 K73 was evolutionarily conserved in different species, implying its important biological function.

We further investigated the role of RAD51 lactylation in the HR repair by performing the DR-GFP reporter assay. OVCAR8-shRAD51 and A2780-shRAD51 cells were constructed to eliminate the effect of endogenous RAD51. shRAD51 reduced the percentage of GFP^+^ cells compared with blank controls. Furthermore, WT RAD51 significantly increased the percentage of GFP^+^ OVCAR8-shRAD51 cells, which could be markedly abolished by the K73R mutant rather than the K40R mutant (Figure 6I and S9A). In A2780-shRAD51 cells, the RAD51 K73R mutant decreased the percentage of GFP^+^ cells to a greater extent than the K40R mutant, implying the importance of RAD51K73la in the HR repair (Figure S9B).

In the comet assay, the RAD51 K73R mutant remarkably prolonged the tail moment compared to K40R in OVCAR8 (Figure 6J and S9C) and A2780 cells (Figure S9D). Additionally, WT RAD51 impaired the CDDP response compared with the blank vector control. However, the RAD51 K73R mutant could markedly reverse the effect to a much greater degree than the K40R mutant, suggesting the essential role of RAD51K73la in CDDP resistance (Figure 6K and S8G). Moreover, the RAD51 K73R mutant more directly abolished the binding signal between RAD51 and GCN5 than the K40R mutant, indicating that RAD51K73la was potentially regulated by GCN5 (Figure 6L). Additionally, molecular docking exhibited the crystallographic structure in which the RAD51K73la peptide interacted with the catalytic pocket of GCN5 (Figure 6M). Therefore, RAD51K73la facilitated the HR repair and conferred CDDP resistance, which was possibly catalyzed by GCN5.

### GCN5 inhibitor increased the response to CDDP by impairing H3K9la and RAD51K73la in ovarian cancer

**Figure 7:**
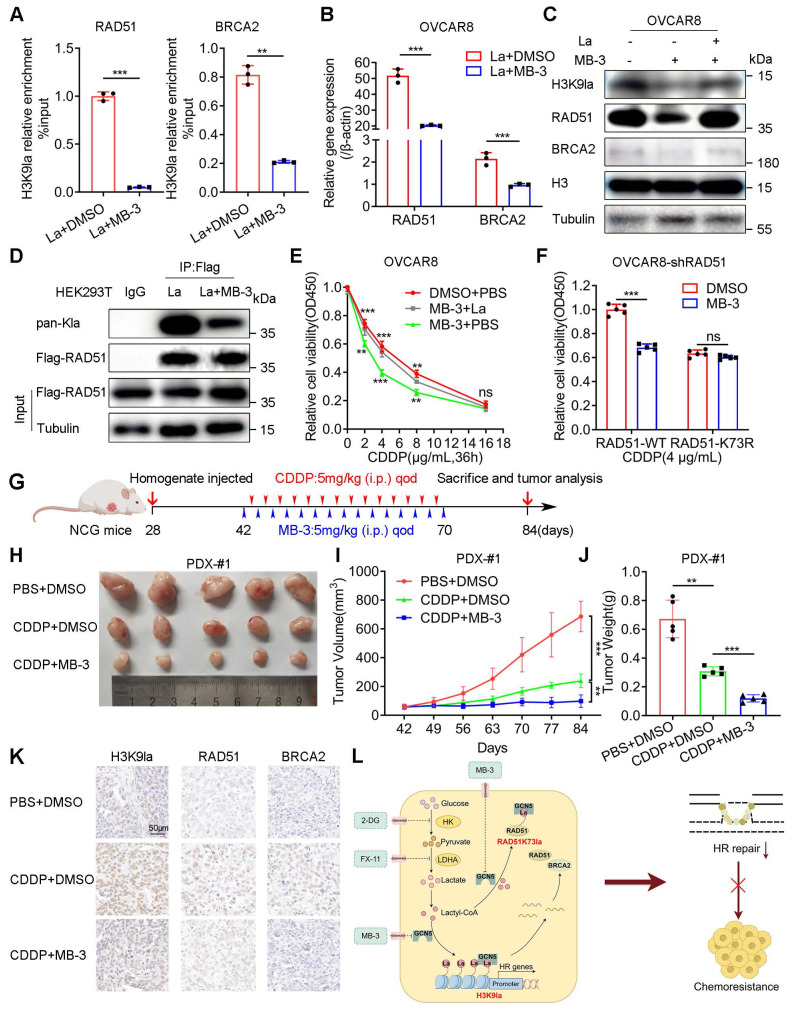
** GCN5 inhibitor MB-3 sensitized ovarian cancer to CDDP by impairing H3K9la and RAD51K73la.** A. ChIP-qPCR analysis of H3K9la-targeted genes in OVCAR8 treated with MB-3 (30 μM) or DMSO. B. qRT-PCR of mRNA levels of H3K9la-targeted genes in OVCAR8 cells treated with MB-3 (30 μM) or DMSO. C. Regulation of H3K9la level and its downstream genes by MB-3 (30 μM). D. Effect of MB-3 on RAD51 lactylation by co-IP and Western blotting (La:10 mM, MB-3: 30 μM). E. Killing efficiency of CDDP in OVCAR8 cells treated with MB-3 or lactate as indicated (La:10 mM, MB-3: 30 μM) by the CCK8 assay. F. Cell viability in OVCAR8-shRAD51 cells transfected with WT or K73R vectors and exposed to CDDP (MB-3: 30 μM) by the CCK8 assay. G. Diagram of PDX model construction, treatment, and tumor analysis in ovarian cancer. H. Tumor images of differently treated groups in PDX-#1. I. Tumor volumes in different PDX-#1 groups on indicated days. J. Tumor weights in different PDX-#1 groups. K. Evaluation of H3K9la, RAD51, and BRCA2 expression from tumor tissues of Figure 7H by IHC. L. Graphical abstract of the study. * *p* < 0.05; ** *p* < 0.01; *** *p* < 0.001; ns, no significant change.

We selected a GCN5 inhibitor, MB-3 (α-methylene-γ-butyrolactone), to explore its biological effect on the CDDP response of ovarian cancer *in vitro* and *in vivo*. The ChIP-qPCR assay illustrated that MB-3 repressed H3K9la enrichment in promoter regions of RAD51 and BRCA2, downregulating their mRNAs (Figure 7A, 7B, and Figure S10A). Consistently, MB-3 induced protein level changes of H3K9la and its target genes, which could be rescued by lactate (Figure 7C and Figure S10B). MB-3 decreased the co-IP signal intensity between H3K9la and GCN5 by interfering with their interaction (Figure S10C and S10D). Besides histone, RAD51 lactylation was also suppressed by MB-3 as determined by co-IP and Western blotting (Figure 7D). Thus, MB-3 might influence the CDDP response of ovarian cancer.

As expected, MB-3 markedly reduced the relative cell viability when cells were exposed to CDDP, which could be rescued by adding lactate (Figure 7E and Figure S10E). Additionally, MB-3 significantly increased the tumor-killing effect of CDDP in A2780-shRAD51 and OVCAR8-shRAD51 cells transfected with the WT RAD51 vector, the effect of which was attenuated by the RAD51 K73R mutant vector (Figure 7F and Figure S10F).

We constructed three PDX models of ovarian cancer by subcutaneously injecting fresh tumor homogenate into NCG mice to validate the combined therapeutic effect of MB-3 on PDX models. Table S7 provided the clinical characteristics of tissue donors. Two weeks after the transplantation, tumor-bearing NCG mice from the same patients were randomized into three treatment groups (PBS + DMSO, CDDP + DMSO, and CDDP + MB-3). The mice received treatments according to the scheme in which CDDP (5 mg/kg every other day) or MB-3 (5 mg/kg every other day) was injected intraperitoneally for 4 weeks (Figure 7G and Figure S10G). In PDX-#1, tumor volume and weight were decreased in the single CDDP group. However, the combination therapy of CDDP and MB-3 showed the lowest tumor burden (Figure 7H - 7J) with downregulation of H3K9la and its target genes in IHC staining (Figure 7K). Intriguingly, although CDDP did not effectively decrease the tumor volume of PDX-#2, MB-3 remarkedly reversed the primary resistance in the CDDP + MB-3 group (Figure S10H). However, MB-3 did not improve the tumor-killing ability of CDDP in PDX-#3, in which the CDDP + MB-3 group exhibited tumor volume and weight similar to the CDDP + DMSO group (Figure S10I). Therefore, we concluded that MB-3 could increase the sensitivity or even reverse the resistance to CDDP in specific ovarian cancer cases. Nevertheless, elucidation of the underlying mechanisms requires further research. Figure 7L presents the schematic of the molecular mechanisms discussed in this study.

## Discussion

Histone lactylation is generally associated with poor prognosis and malignant behavior of cancer, in which H3K18la has been predominantly researched, while other lysine lactylation sites are rarely reported 29, 41-43. Other histone lysine sites may undergo similar modifications in the presence of tumor-derived lactate. This study identified upregulation of multiple histone lactylation sites that were upregulated in platinum-resistant ovarian cancer, among which the most prominently altered was H3K9la, associated with shorter survival in ovarian cancer patients. Subsequent analysis revealed that H3K9la effectively restored the HR repair ability through the transcriptional activation of core HR pathway genes, mainly RAD51 and BRCA2, thereby suppressing the sensitivity to CDDP. Our study revealed the link between histone lactylation and HR repair, elucidating the molecular mechanism through which lactate enhanced DDR from a novel lactylation perspective.

Besides histone, Kla's association with non-histone proteins has also become a crucial research focus, especially its effect on protein functionality in diverse physiological and pathological processes 44. It has been reported that non-histone PTMs play an essential role in HR repair, e.g., crotonylation 45. Previous studies suggested that MRE11 and NBS1, two crucial HR proteins, are lactylated at K673 and K388, respectively, facilitating HR repair and linking it to cellular metabolism 46, 47.

Besides MRE11 and NBS1, we have shown that another crucial HR protein, RAD51, is lactylated at K73 and K40. Significantly, RAD51 lactylation level was positively correlated with CDDP concentration, implying its potential relation to CDDP resistance, and RAD51K73la remarkably affected the CDDP response by augmenting the HR repair ability. Thus, our findings, together with previous reports (46, 47), demonstrated the ubiquity of lactylation in HR proteins, warranting further research.

Non-histone lactylation is widely researched in multiple tumors and biological processes, including spliceosome function 48, metabolic regulation 27, drug resistance 31, translation elongation 49, and transcriptional activation 50. Thus, lactylation in non-histone proteins involved in numerous biological processes, appears prevalent. Also, from the PTM's perspective, the intriguing effect of lactylation on HR repair provides a novel insight into predicting and/or regulating the HR repair ability.

For targeting lactylation, most current studies have focused on identifying the pertinent enzymes that orchestrate lactate generation as well as lactyl-transferases (writers) that add lactyl-coA to the lysine sites. In this context, glycolysis or lactate transporter alteration can affect lactylation in normal or pathological cells, such as glycolysis enzyme inhibitors and monocarboxylate transporter inhibitors 51. As for the enzymes catalyzing lactylation, it has been established that P300 and class I histone deacetylases play major roles as writers and erasers of protein Kla 24, 35. Mammals have three main families of acyltransferases: P300/CREB-binding protein, MOF, and GCN5-related N-acetyltransferase, all of which are involved in lactylation regulation 25, 49, 52, 53. Besides the classical acyltransferases, recent studies have reported that alanyl-tRNA synthetase1 exhibits dual functionality as a genuine lactyl-transferase 50, 54.

Our study demonstrated that glycolysis inhibitors 2-DG and FX-11 could reduce the intracellular lactate accumulation and suppress H3K9la, which was similar to previous research. We reported GCN5 as the predominant regulator of H3K9la among acyltransferase families. Although Chen *et al.* showed that MRE11K63la was mainly lactylated by CBP instead of GCN5, our study confirmed that RAD51K73, a novel Kla site we identified, was directly regulated by GCN5 46. This could be explained by the fact that different proteinsor lysine sites might be lactylated by various catalyzing enzymes and molecular pathways. These observations prompted us to search for additional, specific, and efficient lactyl-transferases to understand this novel PTM and its regulation comprehensively.

Based on its regulating function, GCN5 was correlated with unfavorable prognosis and platinum resistance in our study. Subsequent analyses have shown that GCN5 abolished the cancer-killing ability of CDDP via downregulating H3K9la and its downstream core HR gene expression. Moreover, GCN5 also accelerated RAD51K73la, which impaired the CDDP response. Intrinsically, H3K9la and RAD51K73la, facilitated by GCN5, augmented the HR repair ability. Furthermore, we constructed PDX models of ovarian cancer which were treated with a combination of a GCN5 inhibitor MB-3 and CDDP. In PDX-#1, MB-3 significantly increased the primary sensitivity to CDDP. Nevertheless, in PDX-#3, adding MB-3 did not sensitize formed tumors to CDDP, indicating the heterogeneity of ovarian cancer. MB-3 somewhat reversed the innate CDDP resistance of PDX-#2. Therefore, developing strategies optimizing the use of GCN5 inhibitors in acquired or primary CDDP-resistant ovarian cancer patients is essential. More PDX cases should be included in the research to increase its trustworthiness, which is the main limitation of this study.

Small-molecule agents or gene therapies for lactate production have been widely investigated in lactylation-associated biological processes. Preclinical studies exploring potential therapeutic approaches have assessed the efficacy of multiple glycolysis enzyme inhibitors 55, 56. Many drugs targeting lactylation involve inhibitors of histone acetyltransferases, and the HDAC family has shown favorable efficacy in multiple malignancies 56. Monoclonal antibodies for Kla sites may also be a good approach for Kla-based clinical treatments. Nevertheless, due to a lack of comprehensive studies of the regulatory mechanisms of lactylation, there is no specific therapeutic agent targeting lactylation yet. The specificity and efficacy of approaches targeting lactylation are challenging due to its involvement in other physiological processes. In summary, the recent exploration of lactylation and its mechanism of action has provided new insights into novel therapeutic strategies, warranting further investigation.

## Conclusions

Our findings showed elevated histone or non-histone lactylation, predominantly in H3K9la, conferred CDDP resistance ovarian cancer patients via transcriptional activation of HR core genes, thereby enhancing HR repair. Likewise, RAD51, one of H3K9la downstream genes, could be lactylated at K73 to augment HR repair and cause drug resistance. H3K9la and RAD51K73la shared the same upstream regulator, GCN5, and targeting GCN5 remarkably increased the CDDP response in cell lines and PDX models. Thus, our study uncovered a crucial role of histone or non-histone lactylation in HR repair and platinum resistance, suggesting that lactylation-associated regulators would be a promising therapeutic target in overcoming platinum resistance of ovarian cancer, benefiting the affected patients.

## Supplementary Material

Supplementary figures and tables.

Supplementary file S1: TCGA samples with platinum status involved in this study.

Supplementary file S2: H3K9la enriched peaks in ChIP-seq.

Supplementary file S3: DDR genes and core HR genes involved in this study.

Supplementary file S4: DDR-associated pathways in GO enrichment analysis.

## Abbreviations

CCK8: Cell Counting Kit-8
CDX: Cell line-derived xenograft
ChIP-seq: Chromatin immunoprecipitation sequencing
CRC: Colorectal cancer
co-IP: Co-Immunoprecipitation
DDR: DNA damage response
GFP: Green fluorescent protein
HGSOC: High-grade serous ovarian cancer
HK: Hexokinase
HR: Homologous recombination
HCC: Hepatocellular carcinoma
IF: Immunofluorescence
IHC: Immunohistochemistry
K-M: Kaplan-Meier
Kla: Lactylation
La: L-Lactate
LDH: Lactate dehydrogenase
OS: Overall survival
pan-Kla: Pan-lysine lactylation
PDX: Patient-derived xenograft
PTMs: Post-translational modifications
qRT-PCR: Quantitative real-time PCR
TCGA: The Cancer Genome Atlas
TSS: Transcription start site

## References

[1] Siegel RL, Miller KD, Wagle NS, Jemal A (2023). Cancer statistics, 2023. CA Cancer J Clin.

[2] Sung H, Ferlay J, Siegel RL, Laversanne M, Soerjomataram I, Jemal A (2021). Global Cancer Statistics 2020: GLOBOCAN Estimates of Incidence and Mortality Worldwide for 36 Cancers in 185 Countries. CA Cancer J Clin.

[3] Peres LC, Cushing-Haugen KL, Kobel M, Harris HR, Berchuck A, Rossing MA (2019). Invasive Epithelial Ovarian Cancer Survival by Histotype and Disease Stage. J Natl Cancer Inst.

[4] Richardson DL, Eskander RN, O'Malley DM (2023). Advances in Ovarian Cancer Care and Unmet Treatment Needs for Patients With Platinum Resistance: A Narrative Review. JAMA Oncol.

[5] Lheureux S, Gourley C, Vergote I, Oza AM (2019). Epithelial ovarian cancer. Lancet.

[6] Colombo N, Sessa C, du Bois A, Ledermann J, McCluggage WG, McNeish I (2019). ESMO-ESGO consensus conference recommendations on ovarian cancer: pathology and molecular biology, early and advanced stages, borderline tumours and recurrent diseasedagger. Ann Oncol.

[7] Pujade-Lauraine E, Banerjee S, Pignata S (2019). Management of Platinum-Resistant, Relapsed Epithelial Ovarian Cancer and New Drug Perspectives. J Clin Oncol.

[8] Galluzzi L, Senovilla L, Vitale I, Michels J, Martins I, Kepp O (2012). Molecular mechanisms of cisplatin resistance. Oncogene.

[9] Tang C, Livingston MJ, Safirstein R, Dong Z (2023). Cisplatin nephrotoxicity: new insights and therapeutic implications. Nat Rev Nephrol.

[10] Pilie PG, Tang C, Mills GB, Yap TA (2019). State-of-the-art strategies for targeting the DNA damage response in cancer. Nat Rev Clin Oncol.

[11] Jeggo PA, Pearl LH, Carr AM (2016). DNA repair, genome stability and cancer: a historical perspective. Nat Rev Cancer.

[12] Roos WP, Thomas AD, Kaina B (2016). DNA damage and the balance between survival and death in cancer biology. Nat Rev Cancer.

[13] Hanahan D, Weinberg RA (2011). Hallmarks of cancer: the next generation. Cell.

[14] Cancer Genome Atlas Research N (2011). Integrated genomic analyses of ovarian carcinoma. Nature.

[15] Weigelt B, Comino-Mendez I, de Bruijn I, Tian L, Meisel JL, Garcia-Murillas I (2017). Diverse BRCA1 and BRCA2 Reversion Mutations in Circulating Cell-Free DNA of Therapy-Resistant Breast or Ovarian Cancer. Clin Cancer Res.

[16] Sakai W, Swisher EM, Karlan BY, Agarwal MK, Higgins J, Friedman C (2008). Secondary mutations as a mechanism of cisplatin resistance in BRCA2-mutated cancers. Nature.

[17] Kondrashova O, Nguyen M, Shield-Artin K, Tinker AV, Teng NNH, Harrell MI (2017). Secondary Somatic Mutations Restoring RAD51C and RAD51D Associated with Acquired Resistance to the PARP Inhibitor Rucaparib in High-Grade Ovarian Carcinoma. Cancer Discov.

[18] Pellegrino B, Herencia-Ropero A, Llop-Guevara A, Pedretti F, Moles-Fernandez A, Viaplana C (2022). Preclinical In Vivo Validation of the RAD51 Test for Identification of Homologous Recombination-Deficient Tumors and Patient Stratification. Cancer Res.

[19] Hoppe MM, Jaynes P, Wardyn JD, Upadhyayula SS, Tan TZ, Lie S (2021). Quantitative imaging of RAD51 expression as a marker of platinum resistance in ovarian cancer. EMBO Mol Med.

[20] Lee JM, Hammaren HM, Savitski MM, Baek SH (2023). Control of protein stability by post-translational modifications. Nat Commun.

[21] Pan RY, He L, Zhang J, Liu X, Liao Y, Gao J (2022). Positive feedback regulation of microglial glucose metabolism by histone H4 lysine 12 lactylation in Alzheimer's disease. Cell Metab.

[22] Li X, Yang Y, Zhang B, Lin X, Fu X, An Y (2022). Lactate metabolism in human health and disease. Signal Transduct Target Ther.

[23] Su J, Zheng Z, Bian C, Chang S, Bao J, Yu H (2023). Functions and mechanisms of lactylation in carcinogenesis and immunosuppression. Front Immunol.

[24] Zhang D, Tang Z, Huang H, Zhou G, Cui C, Weng Y (2019). Metabolic regulation of gene expression by histone lactylation. Nature.

[25] Wang N, Wang W, Wang X, Mang G, Chen J, Yan X (2022). Histone Lactylation Boosts Reparative Gene Activation Post-Myocardial Infarction. Circ Res.

[26] Cui H, Xie N, Banerjee S, Ge J, Jiang D, Dey T (2021). Lung Myofibroblasts Promote Macrophage Profibrotic Activity through Lactate-induced Histone Lactylation. Am J Respir Cell Mol Biol.

[27] Yu J, Chai P, Xie M, Ge S, Ruan J, Fan X (2021). Histone lactylation drives oncogenesis by facilitating m(6)A reader protein YTHDF2 expression in ocular melanoma. Genome Biol.

[28] Yang Z, Yan C, Ma J, Peng P, Ren X, Cai S (2023). Lactylome analysis suggests lactylation-dependent mechanisms of metabolic adaptation in hepatocellular carcinoma. Nat Metab.

[29] Li W, Zhou C, Yu L, Hou Z, Liu H, Kong L (2024). Tumor-derived lactate promotes resistance to bevacizumab treatment by facilitating autophagy enhancer protein RUBCNL expression through histone H3 lysine 18 lactylation (H3K18la) in colorectal cancer. Autophagy.

[30] Yang K, Fan M, Wang X, Xu J, Wang Y, Tu F (2022). Lactate promotes macrophage HMGB1 lactylation, acetylation, and exosomal release in polymicrobial sepsis. Cell Death Differ.

[31] Sun L, Zhang Y, Yang B, Sun S, Zhang P, Luo Z (2023). Lactylation of METTL16 promotes cuproptosis via m(6)A-modification on FDX1 mRNA in gastric cancer. Nat Commun.

[32] Xiong J, He J, Zhu J, Pan J, Liao W, Ye H (2022). Lactylation-driven METTL3-mediated RNA m(6)A modification promotes immunosuppression of tumor-infiltrating myeloid cells. Mol Cell.

[33] Gu J, Zhou J, Chen Q, Xu X, Gao J, Li X (2022). Tumor metabolite lactate promotes tumorigenesis by modulating MOESIN lactylation and enhancing TGF-beta signaling in regulatory T cells. Cell Rep.

[34] Wang X, Fan W, Li N, Ma Y, Yao M, Wang G (2023). YY1 lactylation in microglia promotes angiogenesis through transcription activation-mediated upregulation of FGF2. Genome Biol.

[35] Moreno-Yruela C, Zhang D, Wei W, Baek M, Liu W, Gao J (2022). Class I histone deacetylases (HDAC1-3) are histone lysine delactylases. Sci Adv.

[36] Zu H, Li C, Dai C, Pan Y, Ding C, Sun H (2022). SIRT2 functions as a histone delactylase and inhibits the proliferation and migration of neuroblastoma cells. Cell Discov.

[37] Jin J, Bai L, Wang D, Ding W, Cao Z, Yan P (2023). SIRT3-dependent delactylation of cyclin E2 prevents hepatocellular carcinoma growth. EMBO Rep.

[38] Wang T, Ye Z, Li Z, Jing DS, Fan GX, Liu MQ (2023). Lactate-induced protein lactylation: A bridge between epigenetics and metabolic reprogramming in cancer. Cell Prolif.

[39] Sun C, Cao W, Qiu C, Li C, Dongol S, Zhang Z (2020). MiR-509-3 augments the synthetic lethality of PARPi by regulating HR repair in PDX model of HGSOC. J Hematol Oncol.

[40] Knijnenburg TA, Wang L, Zimmermann MT, Chambwe N, Gao GF, Cherniack AD (2018). Genomic and Molecular Landscape of DNA Damage Repair Deficiency across The Cancer Genome Atlas. Cell Rep.

[41] Xie B, Lin J, Chen X, Zhou X, Zhang Y, Fan M (2023). CircXRN2 suppresses tumor progression driven by histone lactylation through activating the Hippo pathway in human bladder cancer. Mol Cancer.

[42] He Y, Ji Z, Gong Y, Fan L, Xu P, Chen X (2023). Numb/Parkin-directed mitochondrial fitness governs cancer cell fate via metabolic regulation of histone lactylation. Cell Rep.

[43] Li F, Zhang H, Huang Y, Li D, Zheng Z, Xie K (2024). Single-cell transcriptome analysis reveals the association between histone lactylation and cisplatin resistance in bladder cancer. Drug Resist Updat.

[44] Gao X, Pang C, Fan Z, Wang Y, Duan Y, Zhan H (2024). Regulation of newly identified lysine lactylation in cancer. Cancer Lett.

[45] Yu H, Bu C, Liu Y, Gong T, Liu X, Liu S (2020). Global crotonylome reveals CDYL-regulated RPA1 crotonylation in homologous recombination-mediated DNA repair. Sci Adv.

[46] Chen Y, Wu J, Zhai L, Zhang T, Yin H, Gao H (2024). Metabolic regulation of homologous recombination repair by MRE11 lactylation. Cell.

[47] Chen H, Li Y, Li H, Chen X, Fu H, Mao D (2024). NBS1 lactylation is required for efficient DNA repair and chemotherapy resistance. Nature.

[48] Yang D, Yin J, Shan L, Yi X, Zhang W, Ding Y (2022). Identification of lysine-lactylated substrates in gastric cancer cells. iScience.

[49] Xie B, Zhang M, Li J, Cui J, Zhang P, Liu F (2024). KAT8-catalyzed lactylation promotes eEF1A2-mediated protein synthesis and colorectal carcinogenesis. Proc Natl Acad Sci U S A.

[50] Zong Z, Xie F, Wang S, Wu X, Zhang Z, Yang B (2024). Alanyl-tRNA synthetase, AARS1, is a lactate sensor and lactyltransferase that lactylates p53 and contributes to tumorigenesis. Cell.

[51] American Association for Cancer Research (2015). New Compound Targets Warburg Effect. Cancer Discov.

[52] Yang Z, Zheng Y, Gao Q (2024). Lysine lactylation in the regulation of tumor biology. Trends Endocrinol Metab.

[53] Niu Z, Chen C, Wang S, Lu C, Wu Z, Wang A (2024). HBO1 catalyzes lysine lactylation and mediates histone H3K9la to regulate gene transcription. Nat Commun.

[54] Ju J, Zhang H, Lin M, Yan Z, An L, Cao Z (2024). The alanyl-tRNA synthetase AARS1 moonlights as a lactyltransferase to promote YAP signaling in gastric cancer. J Clin Invest.

[55] Zhang Y, Song H, Li M, Lu P (2024). Histone lactylation bridges metabolic reprogramming and epigenetic rewiring in driving carcinogenesis: Oncometabolite fuels oncogenic transcription. Clin Transl Med.

[56] Hu XT, Wu XF, Xu JY, Xu X (2024). Lactate-mediated lactylation in human health and diseases: Progress and remaining challenges. J Adv Res.

