# Supplementary material for: Targeting platinum-resistant ovarian cancer by disrupting histone and RAD51 lactylation
Source: Theranostics. 2025 Feb 10;15(7):3055–75. doi: 10.7150/thno.104858 (PMC11898288; doi:10.7150/thno.104858)
Supplement: Supplementary file 1 — Supplementary figures and tables. [file thnov15p3055s1.pdf]

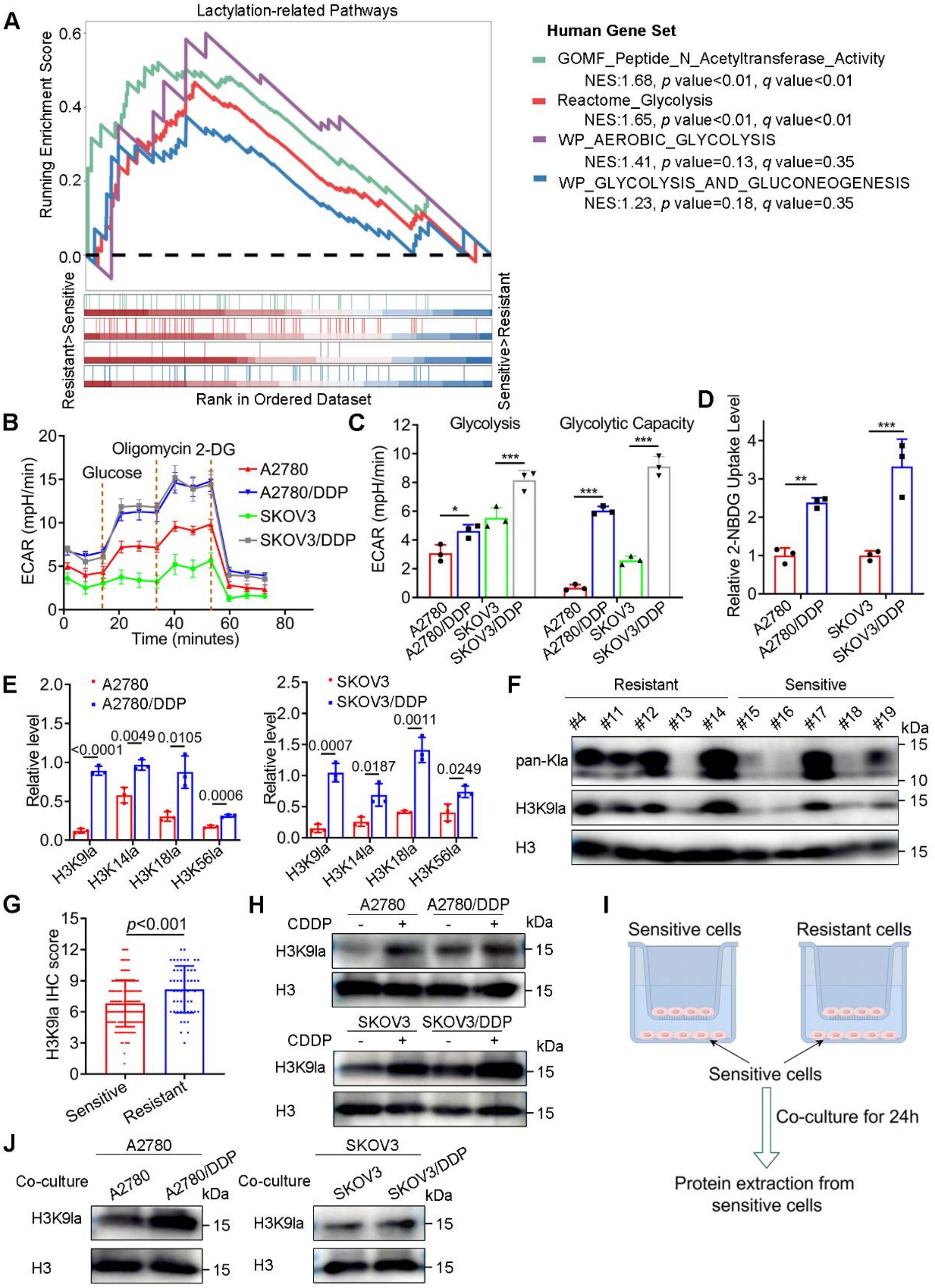

Figure S1. Glycolysis activity and H3K9la level were elevated in platinum-resistant ovarian cancer (related to Figure 1).

A. GSEA analysis of other lactylation-related gene sets in the TCGA cohort.

B. ECAR was measured between CDDP-resistant and corresponding parental cells.

C. Statistical analysis of glycolysis and glycolytic capacity between CDDP-resistant cells and corresponding parental cells.

D. Glucose uptake measurement in CDDP-resistant and control cells.

E. Western blot quantification of H3K9la, H3K18la, H3K14la, and H3K56la in CDDP-resistant and corresponding parental cells.

F. Western blotting of the samples related to Figure 1D. Patient-#4 was used as the inter-band control sample.

G. H3K9la IHC scores between platinum-resistant and platinum-sensitive ovarian cancer patients from the Qilu Hospital cohort.

H. Detection of H3K9la alteration in CDDP-resistant and -sensitive cells treated with CDDP (1 μg/ml).

I. Schematic representation of the co-culture.

J. Western blot of H3K9la alterations in sensitive cells after co-culturing with cisplatin-resistant or -sensitive cells.

* $p < 0.05$; ** $p < 0.01$; *** $p < 0.001$; ns, no significant change.

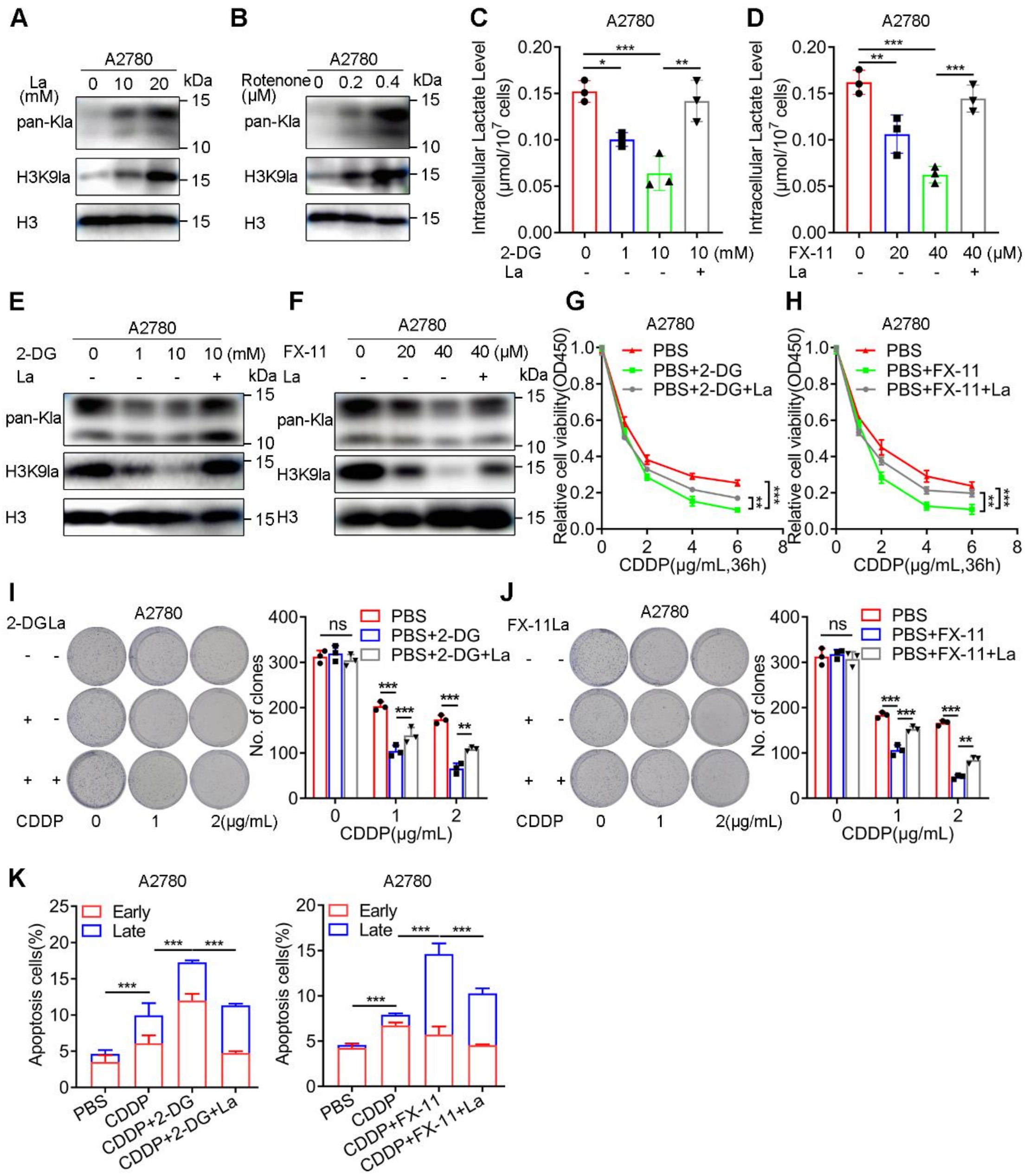

Figure S2. Glycolysis inhibitors decreased H3K9la and sensitized ovarian cancer cells to CDDP (related to Figure 2).

A and B. pan-Kla or H3K9la levels in A2780 cells treated with lactate (A) or rotenone (B) by Western blotting.

C and D. Intracellular lactate levels of A2780 cells treated with 2-DG (C) or FX-11 (D).

E and F. pan-Kla or H3K9la levels regulated by 2-DG (E), FX-11 (F) or La (10 mM) in A2780 cells.

G and H. 2-DG (1 mM) (G) or FX-11 (20 μM) (H) impaired the relative cell viability of A2780 cells exposed to CDDP in the CCK8 assay, which was reversely induced by La (10 mM).

I and J. Colony formation assay of A2780 cells pretreated with 2-DG (1 mM) (I), FX-11 (20 μM) (J), or La (10 mM) when exposed to CDDP.

K and L. 2-DG (1 mM) (K) or FX-11 (20 μM) (L) increased the apoptotic cell ratio (%) induced by CDDP (2 μg/ml), which was reversed by La (10 mM) in A2780 cells.

* $p < 0.05$; ** $p < 0.01$; *** $p < 0.001$; ns, no significant change.

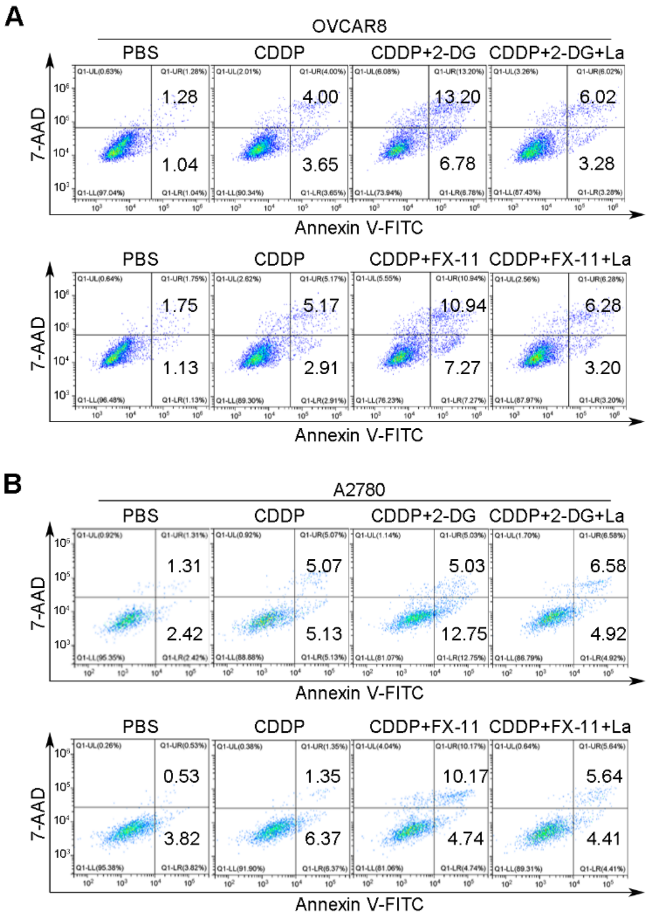

Figure S3. Glycolysis inhibitors promoted CDDP-induced apoptosis (related to Figure 2).

A and B. Flow cytometry to sort apoptotic cells from OVCAR8 (A) or A2780 (B) cells treated with 2-DG (1 mM), FX-11 (20 μM), or La (10 mM) when cells were exposed to CDDP.

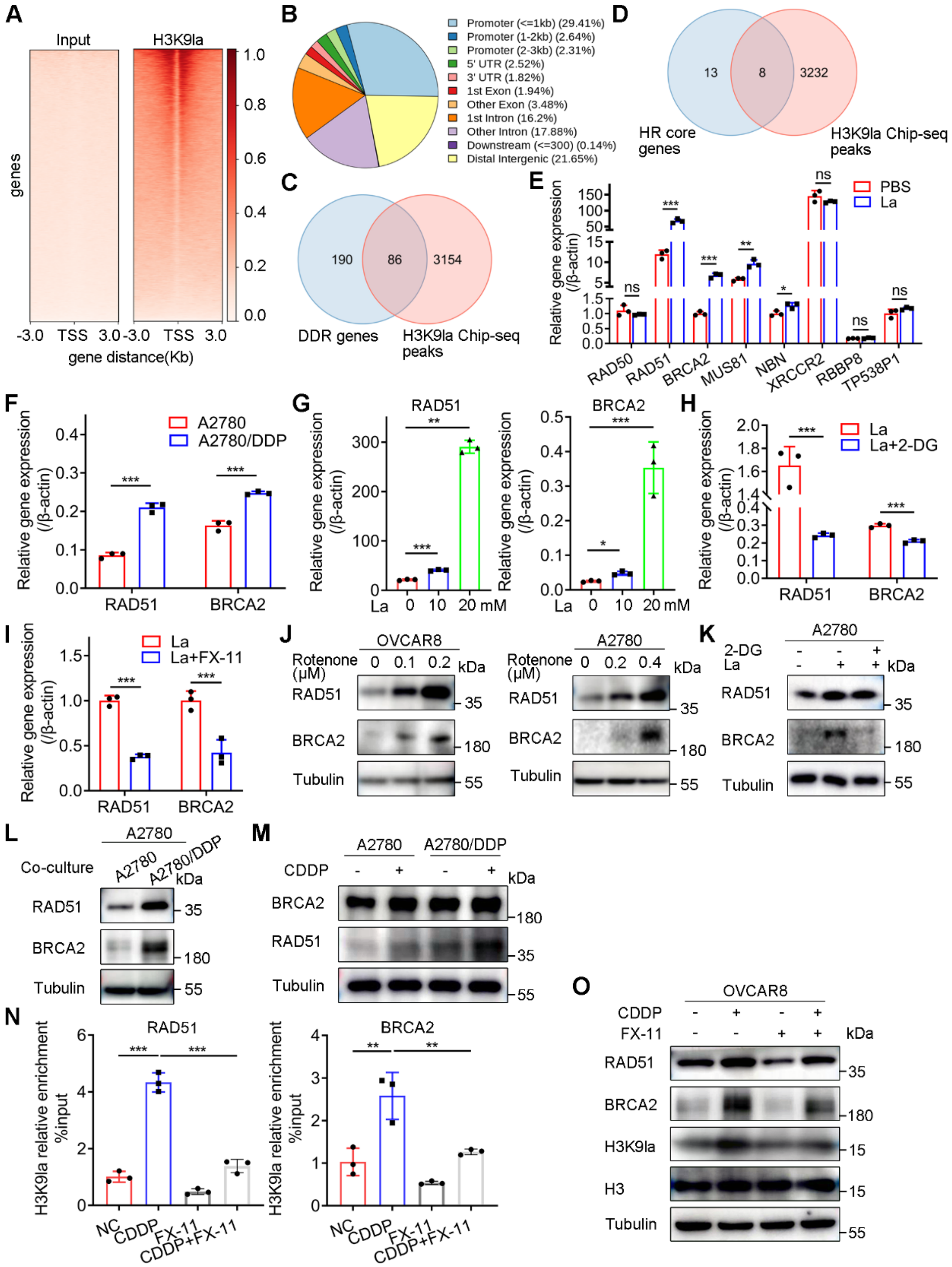

Figure S4. H3K9la activated expression of RAD51 and BRCA2 (related to Figure 3).

A. Visualization of the H3K9la binding density between input and H3K9la samples. The heatmap shows the ChIP-enriched genes and the peak location.

B. Binding peak distribution and proportion in the ChIP-seq assay by anti-H3K9la.

C and D. Venn diagram between H3K9la immunoprecipitated genes and DDR (C) or core HR repair genes (D).

E. qRT-PCR was used to verify the level of candidate genes induced by La (10 mM).

F. mRNA levels of H3K9la target genes in A2780/DDP or control cells by qRT-PCR.

G. mRNA levels of H3K9la target genes in OVCAR8 cells treated with lactate.

H and I. mRNA levels of H3K9la target genes in OVCAR8 cells treated with 2-DG (1 mM) (H), FX-11 (20 μM) (I) or La (10 mM).

J. Rotenone increased RAD51 and BRCA2 protein levels as shown by Western blotting.

K. Western blots of RAD51 and BRCA2 in A2780 cells treated with 2-DG (1 mM) or lactate (10 mM).

L. Detection of RAD51 and BRCA2 alterations in sensitive cells during co-culture with CDDP-resistant and -sensitive cells.

M. Detection of RAD51 and BRCA2 alteration in CDDP-resistant and -sensitive cells treated with CDDP (1 μg/ml).

N. ChIP-qPCR of the promoters in RAD51 and BRCA2 using anti-H3K9la (CDDP: 2 μg/ml, 2-DG: 1 mM, FX-11: 20 μM).

O. Western blotting of RAD51 and BRCA2 in OVCAR8 cells treated with CDDP (2 μg/ml) or FX-11 (20 μM).

* $p < 0.05$; ** $p < 0.01$; *** $p < 0.001$; ns, no significant change.

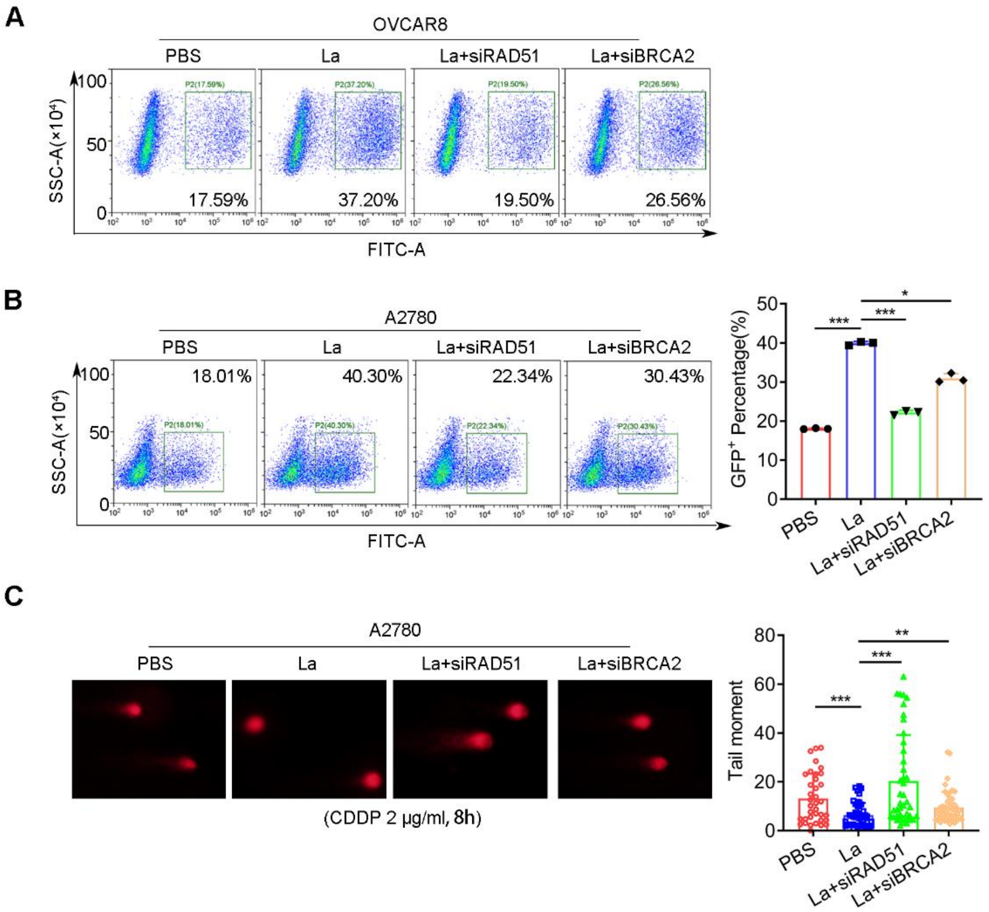

Figure S5. H3K9la facilitated HR repair by increasing RAD51 and BRCA2 (related to Figure 3).

A and B. Fluorescence-activated cell sorting of OVCAR8 (A) and A2780 (B) cells by flow cytometry. Green boxes indicate GFP^+ cells.

C. Comet assay (left) and quantification (right) of A2780 cells treated as indicated (La: 10 mM).

* p < 0.05; ** p < 0.01; *** p < 0.001; ns, no significant change.

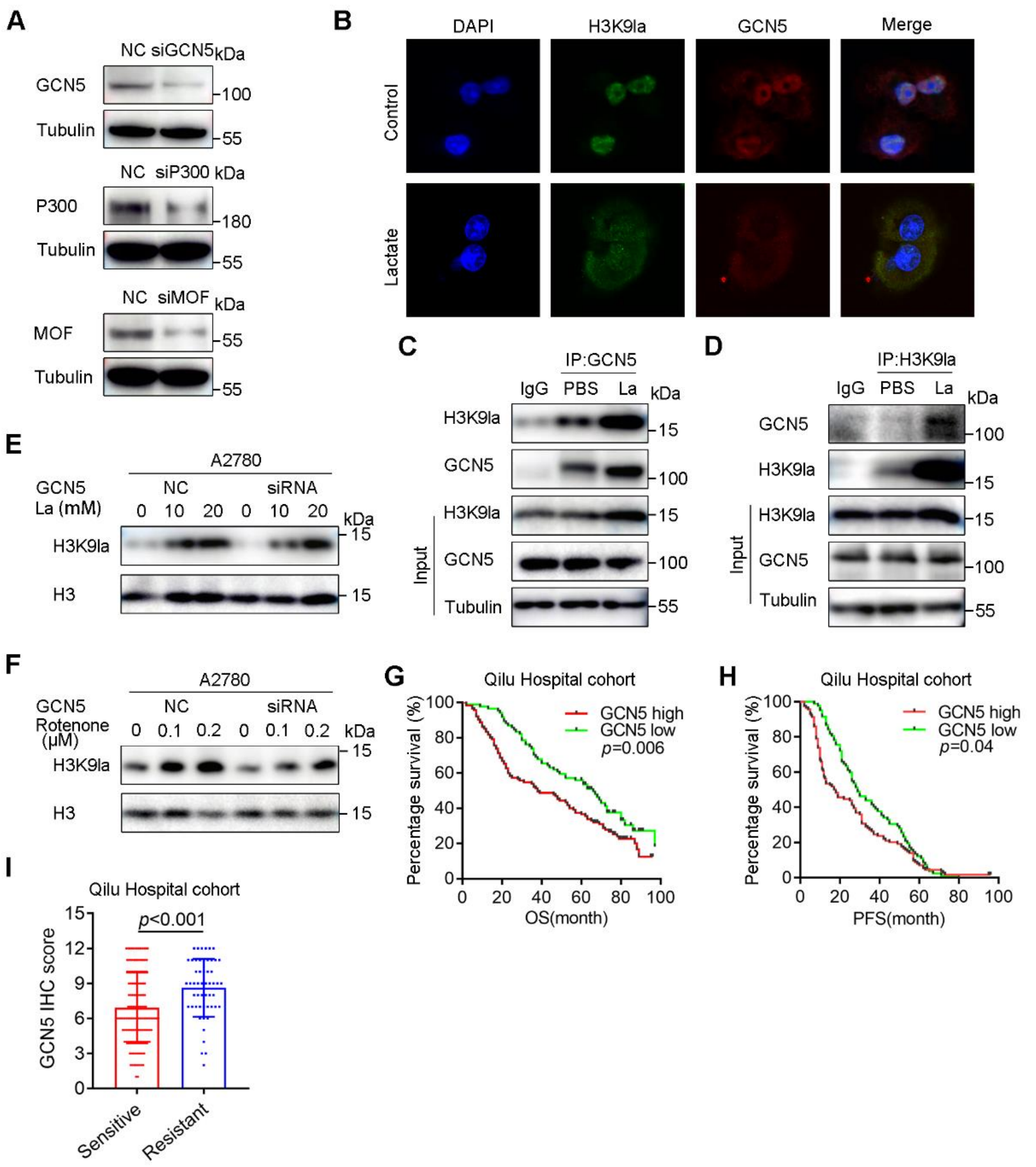

Figure S6. GCN5 positively regulated H3K9la and predicted poor prognosis and platinum resistance (related to Figure 4).

A. Validation of GCN5, P300, and MOF siRNA knockdown efficiency by Western blotting.

B. Immunofluorescence co-staining for H3K9la and GCN5 in OVCAR8 cells.

C and D. Interactions between H3K9la and GCN5 revealed by co-IP analyzed by Western blotting (La: 10 mM).

E and F. H3K9la levels by Western blotting in A2780 cells transfected with GCN5 siRNA or control when cells were treated with lactate (E) or rotenone (F).

G and H. OS (G) and PFS (H) of GCN5 high- and low-expression groups in the Qilu cohort.

I. GCN5 IHC scores of platinum-sensitive and -resistant patients in the Qilu cohort.

* $p < 0.05$; ** $p < 0.01$; *** $p < 0.001$; ns, no significant change.

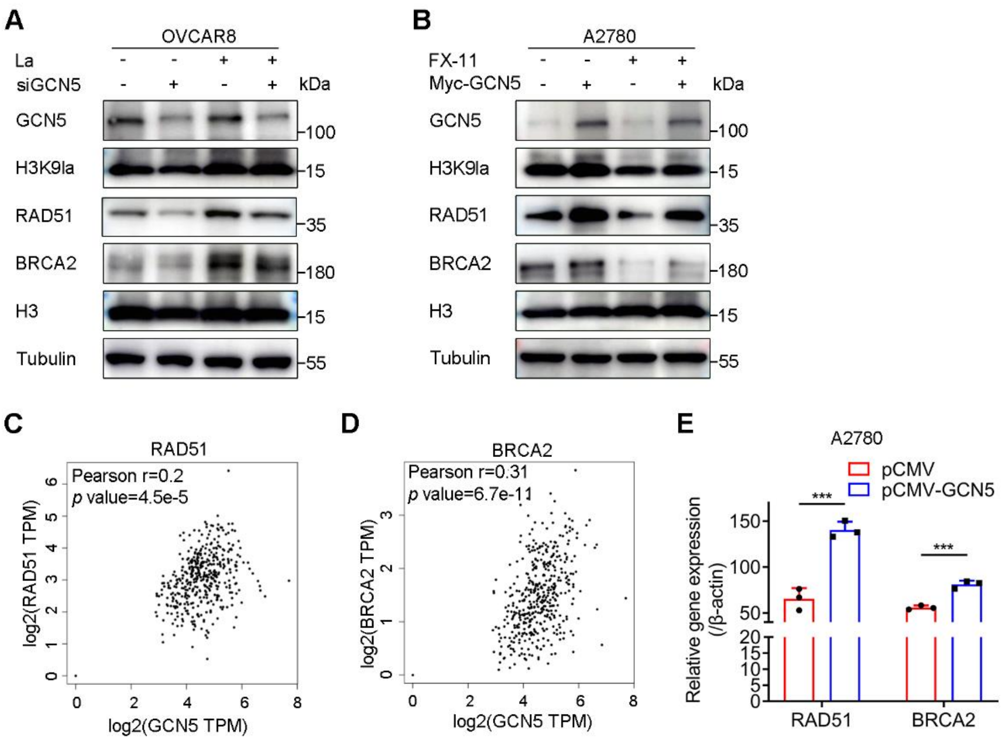

Figure S7. GCN5 positively regulated H3K9la target genes (related to Figure 5).

A. H3K9la and its target gene expression in OVCAR8 cells treated with La (10 mM) or siGCN5.

B. H3K9la and its target gene expression in A2780 cells treated with FX-11 (20 μM) or Myc-GCN5.

C and D. Pearson correlation analysis between GCN5 and RAD51 (C) or BRCA2

(D) in the GEPIA2 database.

E. qRT-PCR analysis of H3K9la target genes in GCN5-overexpressing or control A2780 cells.

* $p < 0.05$; ** $p < 0.01$; *** $p < 0.001$; ns, no significant change.

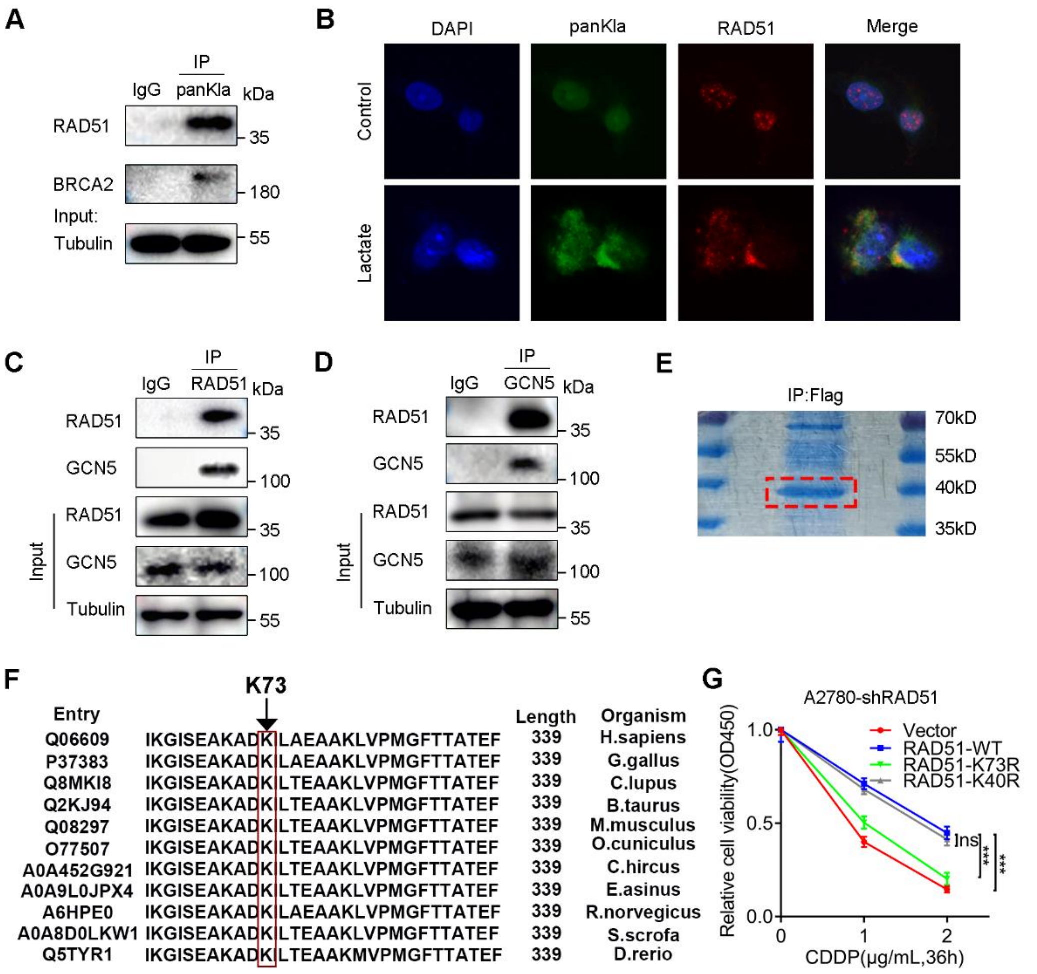

Figure S8. RAD51 was lactylated at K73 (related to Figure 6).

A. Endogenous RAD51 and BRCA2 lactylation by anti-pan-Kla co-IP and Western blotting.

B. Immunofluorescence co-staining of RAD51 and pan-Kla in OVCAR8 cells (La: 10 mM).

C and D. Interaction between GCN5 and RAD51 by anti-RAD51 (C) or anti-GCN5 (D) co-IP and Western blotting.

E. LC-MS/MS analysis of the Coomassie-stained gel section (indicated in the red-dotted-line box).

F. Conservation analysis of K73 in RAD51 across different species.

G. CCK8 assay to detect the cell viability of A2780-shGCN5 cells transfected with WT, K73R, or K40R vectors with different CDDP concentrations.

* $p < 0.05$; ** $p < 0.01$; *** $p < 0.001$; ns, no significant change.

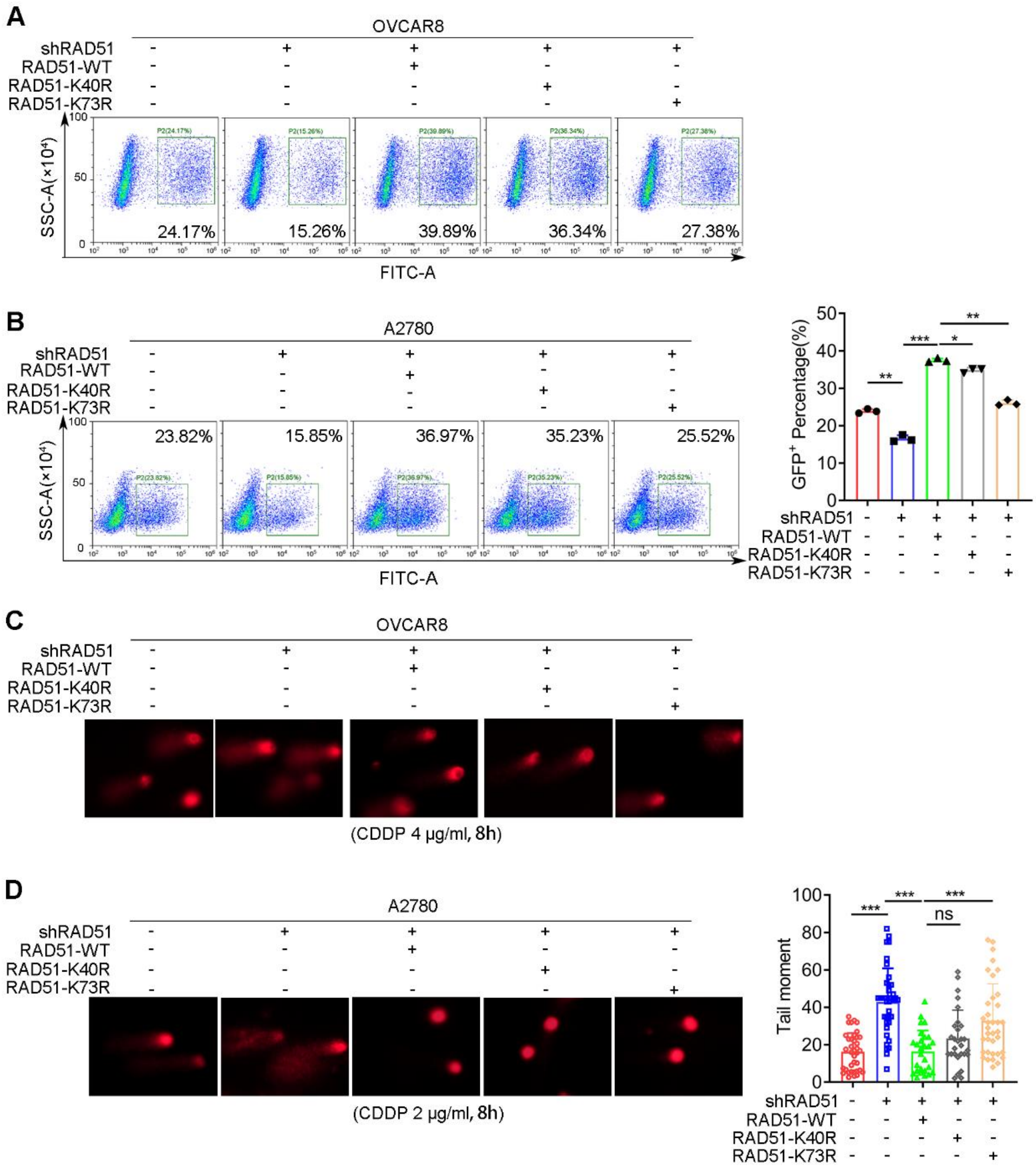

Figure S9. RAD51K73la facilitated HR repair (related to Figure 6).

A. Flow cytometry images related to Figure 6I; green boxes indicate GFP^+ cells.

B. Flow cytometry images (left) and quantification (right) of A2780 cells treated as in Figure 6I; green boxes indicate GFP^+ cells.

C. Comet assay images related to Figure 6J.

D. Comet assay (left) and quantification (right) of A2780 cells treated as in Figure 6J.

* $p < 0.05$; ** $p < 0.01$; *** $p < 0.001$; ns, no significant change.

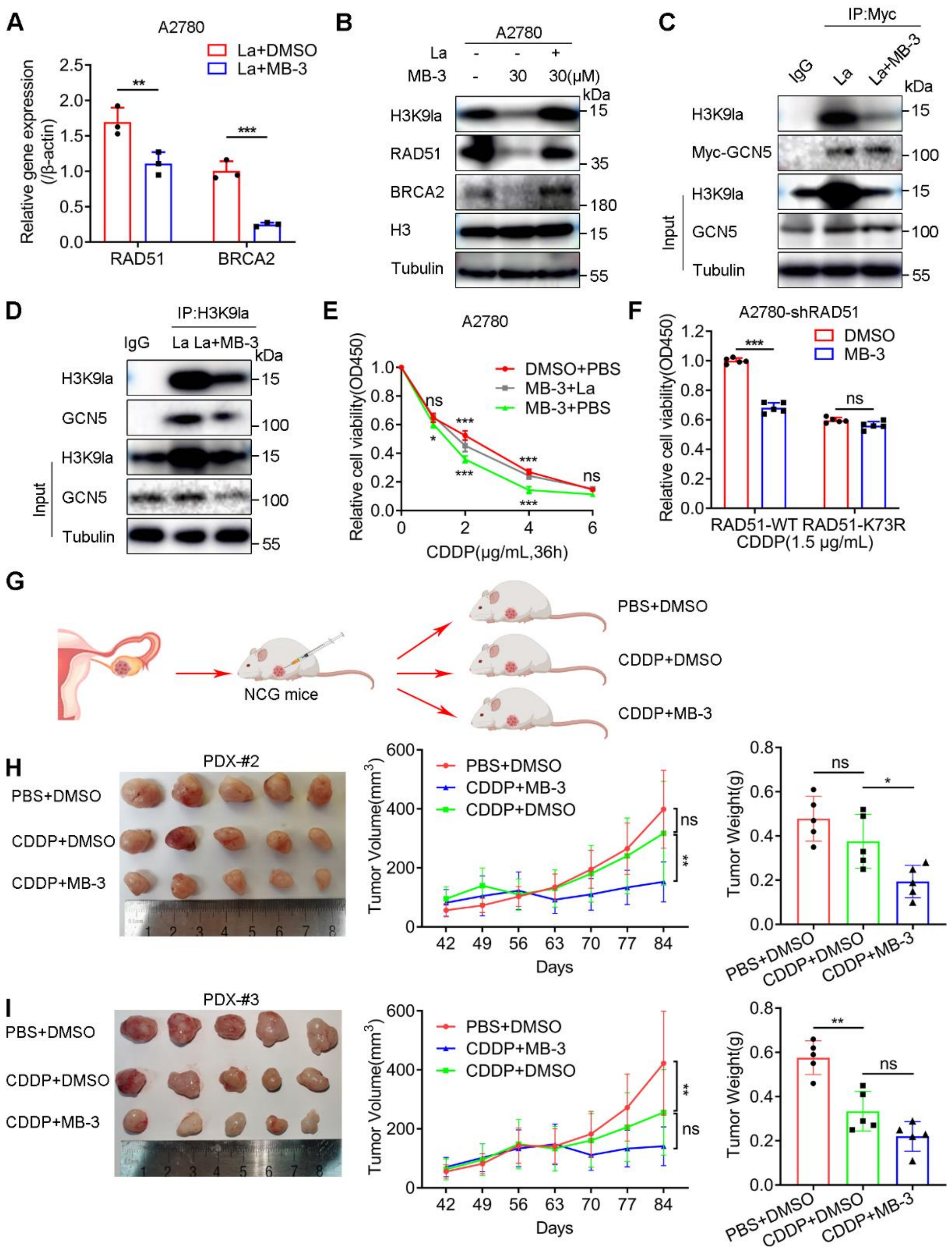

Figure S10. MB-3 increased response to CDDP by impairing H3K9la and RAD51K73la in ovarian cancer cells and PDX models (related to Figure 7).

A. qRT-PCR assay to detect H3K9la target genes in A2780 cells treated with MB-3 (30 μM).

B. H3K9la target gene expression in A2780 cells treated with MB-3 (30 μM) by

Western blotting.

C and D. Interaction between H3K9la and GCN5 by anti-Myc (C) or anti-H3K9la (D) co-IP in HEK293T cells treated with MB-3 (30 μM).

E. Killing efficiency of CDDP in A2780 cells treated with MB-3 (30 μM) or lactate (10 mM) by the CCK8 assay.

F. Cell viability of A2780-shRAD51 cells transfected with WT or K73R vectors when exposed to CDDP and MB-3 (30 μM) by the CCK8 assay.

G. Construction and grouping of PDX models for the MB-3 treatment assay.

H and I. Tumor images, volume, and weight of PDX-#2 (H) and PDX-#3 (I).

* $p < 0.05$; ** $p < 0.01$; *** $p < 0.001$; ns, no significant change.

Table S1. Lactylation-related Gene Sets

Term	ID	Database
HALLMARK_GLYCOLYSIS	H: Hallmark	Arthur Liberzon
REACTOME_GLYCOLYSIS	R-HSA-70171	Reactome
WP_GLYCOLYSIS_AND_GLUCONEOGENESIS	WP534	Wiki Pathways
GOMF_PEPTIDE_N_ACETYLTRANSFERASE_ACTIVITY	GO: 0034212	Gene Ontology
WP_AEROBIC_GLYCOLYSIS	WP4629	Wiki Pathways

Table S2. Correlation between H3K91a level and Clinical Characteristics

**Clinicopathological features**		**H3K9la expression**			
		Low	High	*p* value	χ^2
**Age (years)**	<56	45	53	0.3813	0.7665
	≥56	41	62		
**FIGO Stage**	I+II	17	11	0.0388	4.271
	III+IV	69	104		
**Platinum Status**	resistant	15	38	0.0107	6.514
	sensitive	70	74		

Table S3. H3K9la ChIP Enriched DDR-related KEGG Pathways

Term	ID	Count	*p* value
DNA replication	hsa03030	13	0.006175222
Base excision repair	hsa03410	15	0.006262819
Nucleotide excision repair	hsa03420	19	0.009978262
Homologous recombination	hsa03440	13	0.020012171
Fanconi anemia pathway	hsa03460	15	0.042170356
Mismatch repair	hsa03430	7	0.094627752

Table S4. Primers used in qRT-PCR

Gene	Forward	Reverse
RAD50	GGAAGAGCAGTTGTCCAGTTACG	GAGTAAACTGCTGTGGCTCCAG
RAD51	TCTCTGGCAGTGATGTCCTGGA	TAAAGGGCGGTGGCACTGTCTA

NBN	TCTGTCAGGACGGCAG GAAAGA	CACCTCCAAAGACAACT GCGGA
TP53BP1	AAGCCAGGCAAGAGAA TGAGGC	GGCTGTTGACTCTGCCT GATTG
XRCC2	TCTGTTTGCTGATGAA GATTCACC	CATCGTGCTGTTAGGTG ATAAAGC
BRCA2	GGCTTCAAAAAGCACT CCAGATG	GGATTCTGTATCTCTTGA CGTTCC
MUS81	GATCCTACAGCACTTC GGAGAC	AAGAGTCCTGGACTTCC GCAAG
RBBP8	TGGCAGACAGTTTCTC CCAAGC	GGCTCCACAAACGCTTT CTGCT
β-actin	CACCATTGGCAATGAG CGGTTC	AGGTCTTTGCGGATGTC CACGT

Table S5. Primers sequence in ChIP-qPCR validation

Target Gene	Forward	Reverse
RAD51	AGTCTGTAAACTCGCG CAGGA	GTAACGTATCCCCGCCT CCC
BRCA2	TGTAAGATCGGCTCGC TTTGG	GACACGACCTGGGACC TATGA

Table S6. Sequences for siRNA

Target Gene	Sequence (5’ to 3’)	
	1	2
RAD51	AAGCUGAAGCUAUGUU CGCCA	GAGCUUGACAAACUAC UUC
BRCA2	CAGGACACAAUUACAA CUAAA	AACAACAAUUACGAACC AAACUU
GCN5	GCAACUCUCUGACGC CCAATT	-
P300	GGAUUCGUCUGUAUG GCUGGUUUAA	-
MOF	AAAGACCAUAAGA UUUAUU	-

Table S7. Characteristics of Patients Enrolled in PDX models

Patient No.	Diagnosis Age	FIGO Stage	Histology	NACT	Sample Site
PDX-#1	54	IIIC	HGSOC	No	Ovary
PDX-#2	59	IIIC	HGSOC	No	Ovary
PDX-#3	62	IIIC	HGSOC	No	Ovary

Table S8. Antibodies Used in This Study

Antibodies or Reagents	Source	Identifier
Rabbit monoclonal anti-Flag Tag	Abcam	Cat#ab205606
Mouse monoclonal anti-Myc Tag	Abcam	Cat#ab32
Rabbit polyclonal anti-H3	Abcam	Cat#ab1791
Mouse monoclonal Anti-alpha Tubulin	Abcam	Cat#ab7291
Anti-rabbit IgG, HRP-linked Antibody	Cell Signaling Technology	Cat#7074
Anti-mouse IgG, HRP-linked Antibody	Cell Signaling Technology	Cat#7076
VeriBlot IP Detection Reagent (HRP)	Abcam	Cat#ab131366
Rabbit monoclonal Anti-L-Lactyl Lysine	PTM BIO	Cat#PTM-1401RM
Rabbit monoclonal anti-L-Lactyl-Histone H4 (Lys5)	PTM BIO	Cat#PTM-1407RM
Rabbit monoclonal anti-L-Lactyl-Histone H4 (Lys8)	PTM BIO	Cat#PTM-1415RM
Rabbit monoclonal anti-L-Lactyl-Histone H4 (Lys12)	PTM BIO	Cat#PTM-1411 RM
Rabbit monoclonal anti-L-Lactyl-Histone H4 (Lys16)	PTM BIO	Cat#PTM-1417RM
Rabbit monoclonal anti-L-Lactyl-Histone H3 (Lys9)	PTM BIO	Cat#PTM-1419RM
Rabbit monoclonal anti-L-Lactyl-Histone H3 (Lys14)	PTM BIO	Cat#PTM-1414RM
Rabbit monoclonal anti-L-Lactyl-Histone H3 (Lys18)	PTM BIO	Cat#PTM-1406RM
Rabbit monoclonal anti-L-Lactyl-Histone H3 (Lys56)	PTM BIO	Cat#PTM-1421RM
Mouse monoclonal anti-RAD51	Abcam	Cat# ab213

Rabbit polyclonal anti-BRCA2	Proteintech Group	Cat#29450-1-AP
Mouse monoclonal anti-GCN5	Proteintech Group	Cat#28390-1-AP
Rabbit monoclonal anti-MOF	Abcam	Cat#ab200660
Rabbit monoclonal anti-P300	Abcam	Cat#ab275378
